# Dynamic transcriptome and phytohormone profiling along the time of light exposure in the mesocotyl of rice seedling

**DOI:** 10.1038/s41598-017-12326-2

**Published:** 2017-09-20

**Authors:** Fangjun Feng, Hanwei Mei, Peiqing Fan, Yanan Li, Xiaoyan Xu, Haibin Wei, Ming Yan, Lijun Luo

**Affiliations:** Shanghai Agrobiological Gene Center; Shanghai Research Station of Crop Gene Resource & Germplasm Enhancement, Chinese Ministry of Agriculture, Shanghai, 201106 China

## Abstract

Mesocotyl elongation is an important trait influencing seedling emergence and establishment in rice direct-seeding cultivation and is immediately inhibited after light exposure. Detailed researches on the molecular basis and biological processes underlying light repression of mesocotyl growth could probably provide useful information for key factors controlling this trait. Here we monitored the transcriptome and endogenous phytohormone changes specifically in the elongating mesocotyl in response to light exposure with a time-course. It was revealed that 974 transcripts were significantly differentially expressed (FDR < 0.05, |log_2_ (L/D) | ≥2) after light exposure. Most of the differential expression genes associated with the responses to hormone. Metabolic pathway analysis using the KEGG system suggested plant hormone signal transduction, α-linolenic acid metabolism and diterpenoid biosynthesis were critical processes of mesocotyl growth inhibited by light. Consistent with DEGs, the endogenous IAA, tZ and GA_3_ content was significantly reduced while JA level was dramatically increased, which indicated that light inhibited rice mesocotyl growth through decreasing IAA, tZ and GA_3_ content and/or increasing JA level. The present results enriched our knowledge about the genes and phytohormones regulating mesocotyl elongation in rice, which may help improve future studies on associated genes and develop new varieties tolerance to deep sowing.

## Introduction

Transplanting rice in flooded field is the dominant way of rice production in China and many other Asian countries for a long history^1^. To facing the challenge of shortage in both labor and water resources, dry direct seeding, which is a simple, convenient and water saving cultivation technique, has become a popular method in some rice-growing areas^2^. In particular, deep direct seeding improves not only plant lodging resistance, but also water and nutrient uptake from the deep soil layer^3,4^. However, delayed emergence and poor seedling establishment happened in most modern rice varieties which were sensitive to sowing depth. Previous studies reported the positive effect of mesocotyl elongation on rapid seedling establishment, consequently on tolerance to sowing depth and early seedling vigor^5–7^.

Mesocotyl is the organ located between the coleoptilar node and the basal part of the seminal root in the young seedlings. Elongation of both mesocotyl and coleoptile push the shoot tip above the soil surface during germination^8,9^. Mesocotyl elongation in rice is controlled by several genetic factors, developmental and environmental signals, such as light^10,11^ and phytohormone^12–15^. In general, light absorption by phytochrome in plants inhibits mesocotyl growth^10,11,16^. Mesocotyl elongation seems to be promoted by endogenous abscisic acid (ABA)^12^ through increasing the cell division activity of the meristem but inhibited by endogenous jasmonate (JA) in rice^14,17^. Other research showed that strigolactones (SL) negatively regulate mesocotyl elongation by controlling cell division but not cell elongation in rice during germination and growth in the darkness^15^. Rice mutants defective in SL related genes (d3, d10, d14, d17 and d27) produced longer mesocotyl than the wild type when grown in the darkness^15,18^. A transcription factor (*OsTCP5*), belonging to the cell division-regulating TCP family, was regulated by SL and cytokinin (CK) and had expression levels negatively correlated with mesocotyl length^19^.

In this study, we investigated the dynamic changes in transcriptome and phytohormone in the mesocotyl of rice seedling under dark and exposed to light for different time periods. We found significant changes in both expression levels of phytohormone-related genes and the contents of endogenous phytohormones in response to light at one time point or more time points. Furthermore, light inhibition of mesocotyl elongation could be caused by lower functioning of growth enhancing phytohormones (IAA, tZ, GA_3_) and/or higher level of repressing phytohormone (JA). The finding of this study enriched our knowledge about the genes and endogenous phytohormones regulating mesocotyl elongation in rice.

## Results

### Characterization of rice mesocotyl elongation

**Figure 1:**
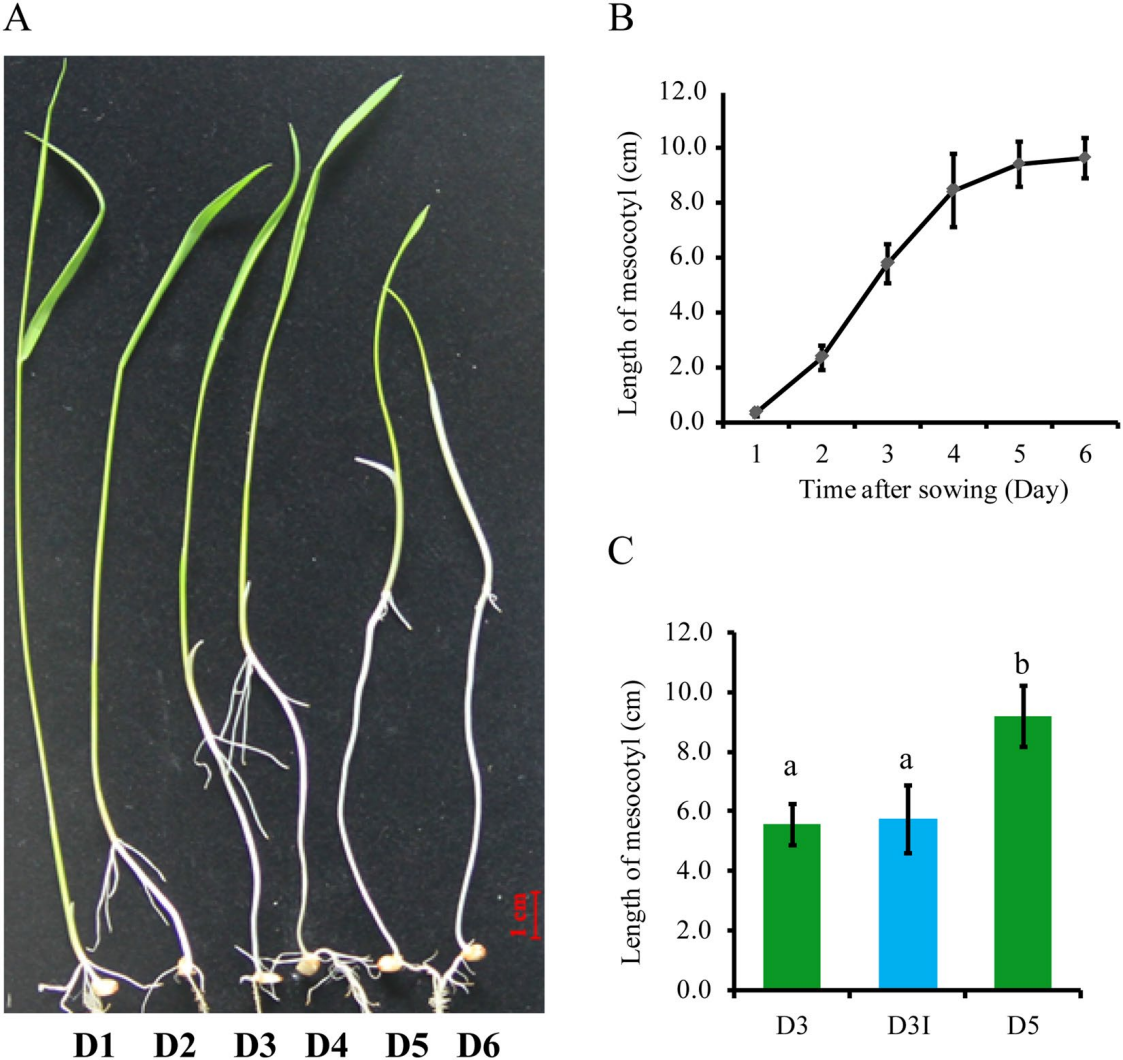
Time-course of mesocotyl elongation of dark-grown ZXM seedlings. (**A**) Seedlings of ZXM exposed to white light since the indicated number of days after sowing; (**B**) Mesocotyl lengths of the seedlings; (**C**) For light interruption experiments, 3-day-old dark-grown ZXM seedlings were irradiated with white light for one minutes and then kept in ongoing darkness for another two days. a and b indicate a significant difference at *P* < 0.01 between the compared pairs. The data represent mean ± SD, n = 10.

Daily measurements of mesocotyl length of Zhaxima (ZXM) seedlings grown in darkness demonstrated that the mesocotyls of the seedlings began to elongate since 1d after sowing and continued to elongate until to 4d at similarly high rates. Mesocotyl elongation then slowed down since 5d after sowing (Fig. 1A, B).

It has been observed that the mesocotyl growth was inhibited when the seedlings came out the soil surface to meet the light. To estimate the sensitivity of mesocotyl elongation to light, 3d dark-grown rice seedlings were exposed to white light (10 μmol/m^2^s) for only one minute and then grown in darkness for another two days (D3I, Fig. 1C). The mesocotyl lengths of D3I seedlings measured at 5d after sowing were almost the same as the mesocotyl lengths measured at 3d after sowing (D3), much shorter than the mesocotyl of seedlings grown under darkness for five days (D5). The results showed that the mesocotyl growth was completely inhibited by a small dosage of light exposure.

### Light-induced transcriptomic changes in rice mesocotyl

**Figure 2:**
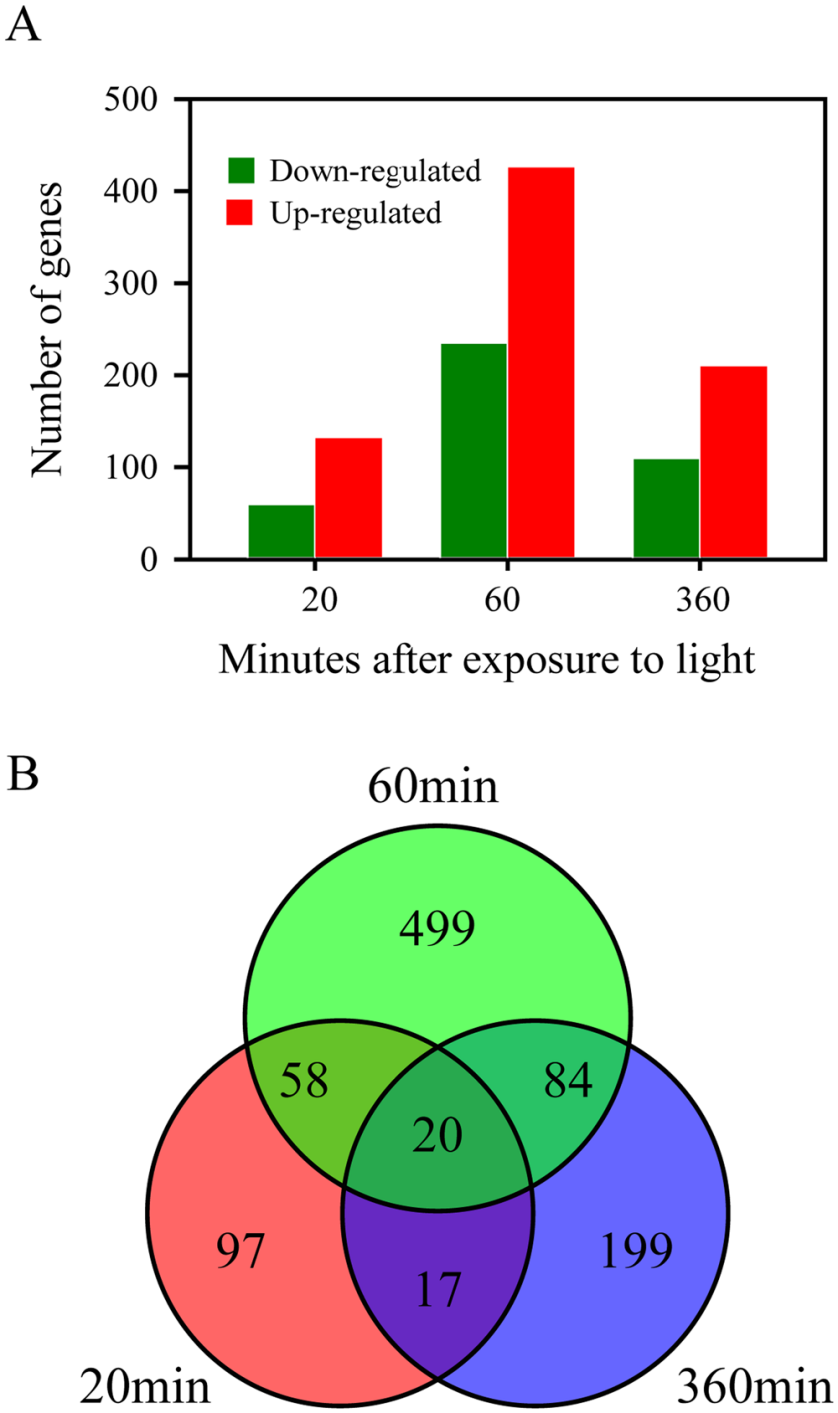
Comparative analysis of transcriptomic changes in rice mesocotyl in response to light at three time-points. (**A**) Number of up-regulated and down-regulated DEGs after light treatment. (**B**) Venn diagram constructed using the DEGs with changes of more than four folds and the FDR values less than 0.05 for at least one time point.

A time-course RNA-Seq experiment was implemented to determine the transcriptomic changes in rice mesocotyl in response to 20 min, 60 min and 360 min light exposure. A total of 23 K to 28 K transcripts were detected at fragments per kilobase of transcript per million mapped reads (FPKM) values ≥ 0.2 in a single replicate under dark or light conditions at three time points (Table S1). Genes with the FDR value < 0.05 and |log_2_ (L/D) | ≥2 were considered to have significantly different expression (DEGs). A total of 974 transcripts were differentially expressed at one time point or more time points (Table S2). The number of DEGs was 192 (132 up- and 60 down-regulated), 661 (426 up- and 235 down-regulated) and 320 (210 up- and 110 down-regulated) at 20 min, 60 min and 360 min light exposure, respectively. It is obvious that 60 min light exposure induced the most extensive changes in gene expression among three time points. At all time points, more genes were up-regulated than down-regulated (Fig. 2A). As shown in the Venn diagram, 20 DEGs were commonly detected in response to three light treatments. Among them, six genes related to phytohormone were validated by qPCR including one genes responding to auxin, two genes responding to gibberellins and five genes responding to jasmonic acid. For most of these genes, expression patterns from qPCR were highly consistent with the results of RNA-seq (Fig. S1). A set of 97 genes were exclusively expressed after 20 min of light exposure that could probably play important roles in the light-induced early signaling events.

### Gene ontology and KEGG pathway enrichment analysis

**Figure 3:**
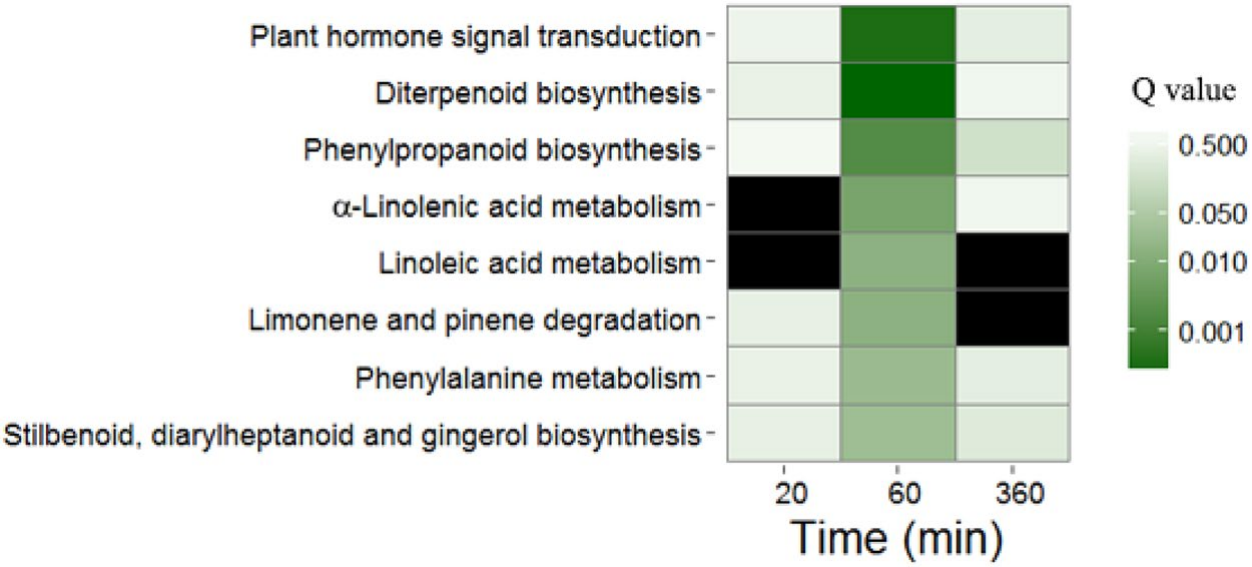
Kyoto Encyclopedia of Genes and Genomes (KEGG) pathway enrichment analysis of the effect of light treatment in rice mesocotyl. KEGG pathway analyses were applied to genes expressed differently in light-treated and untreated samples. The pathways with the Q value (corrected p value, shown in Table S4) of less than 0.05 are shown at least one time point. The Q value determines the degree of color saturation of the corresponding box. The black color indicates that none of the pathway is enriched.

To determine the functional roles of DEGs at each time point, we conducted gene ontology (GO) enrichment analysis using the goatools python package^20^. The numbers of enriched GO terms related to biological process were 31, 84 and 20 at three time points, respectively (Table S3). Among the early-regulated genes (20 min of light exposure), the genes related to regulation of transcription (GO: 0006355) were the most abundant functional term (Table S3), consistent with the major role of transcription factors in regulation of light-responsive gene expression^21^. After 60 min of light exposure, GO analysis revealed a behavior partially similar to that observed at the first time point, further highlighting the involvement of transcription factor in the response of rice seedling to light treatment. Moreover, GO terms for response to light stimulus (GO: 0009416) and response to hormone (GO: 0009725) were also significantly enriched (Table S3). In agreement with previous reports about the major role of hormone in mesocotyl elongation^11,14^, GO analysis evidenced a highly significant overrepresentation of “response to hormone”, “response to jasmonic acid” and “response to salicylic acid”. Among the late-regulated genes (360 min of light exposure), the most overrepresented functional terms were related to “response to hormone” (GO: 0009725) and “response to gibberellins” (GO: 0009739), consistent with that GA was required for auxin-mediated hypocotyls elongation in Arabidopsis^22^. These GO analysis provided valuable clues to investigate the specific processes and functions of transcriptome changes of rice mesocotyl after exposure to light. To obtain a complete view of the significantly altered pathways at the transcriptional level in rice mesocotyl after exposure to light, we performed pathway enrichment analysis for RNA-seq data using the KOBAS^23^. The pathways for hormone signal transduction, diterpenoid biosynthesis and α-linolenic acid metabolism were significantly enriched at 60 min (Fig. 3). Both GO and KEGG analysis showed that hormone signaling and synthesis were significantly enriched, which indicated that phytohormones played an important role in the regulation of mesocotyl elongation.

### Expression patterns of transcription factors in mesocotyl after light exposure

**Figure 4:**
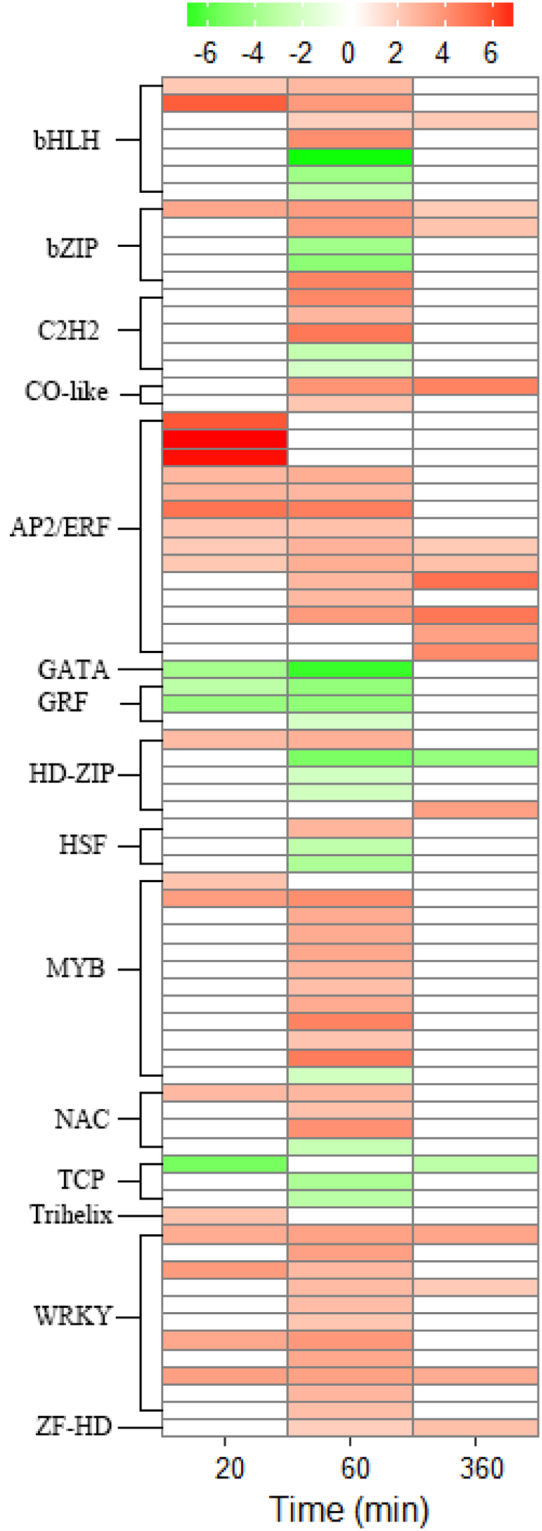
Detailed expression profile of DEGs related to transcription factors at three time points. Heat maps illustrating the relative expression levels of genes encoding transcription factor obtained from MSU Rice Genome Annotation Project Release 7 (http://rice.plantbiology.msu.edu/). Red and green colors depict up- and down-regulation relative to control (light/dark), respectively. The scale shows log2 fold change.

Seventy-seven putative transcription factors (TFs), belonging to 15 families were differentially expressed, 56 up-regulated and 21 down-regulated, in response to light at one or more time points. The number of up-regulated TFs was always higher than that of down-regulated TFs at all three time points. Further, the members of the GRF, TCP and GATA TF family were all strongly down-regulated at the early stage of light induction. In contrast, members of AP2/ERF, WRKY, CO-like and ZF-HD TF family were all up-regulated (Fig. 4) at one time point or more points. Among the up-regulated genes, the largest family was AP2/ERF (14 genes), followed by WRKY (11 genes). A bZIP family gene LOC_Os01g07880, having a homologue (HY5) regulating hypocotyl elongation by light in Arabidopsis^24^, exhibited increased levels of expression at all three time points (Table S4). Nevertheless, solid genetic evidence is required to validate whether these TFs are directly involved in the regulation of mesocotyl elongation by light.

### Expression profiles of genes associated with phytohormones

**Figure 5:**
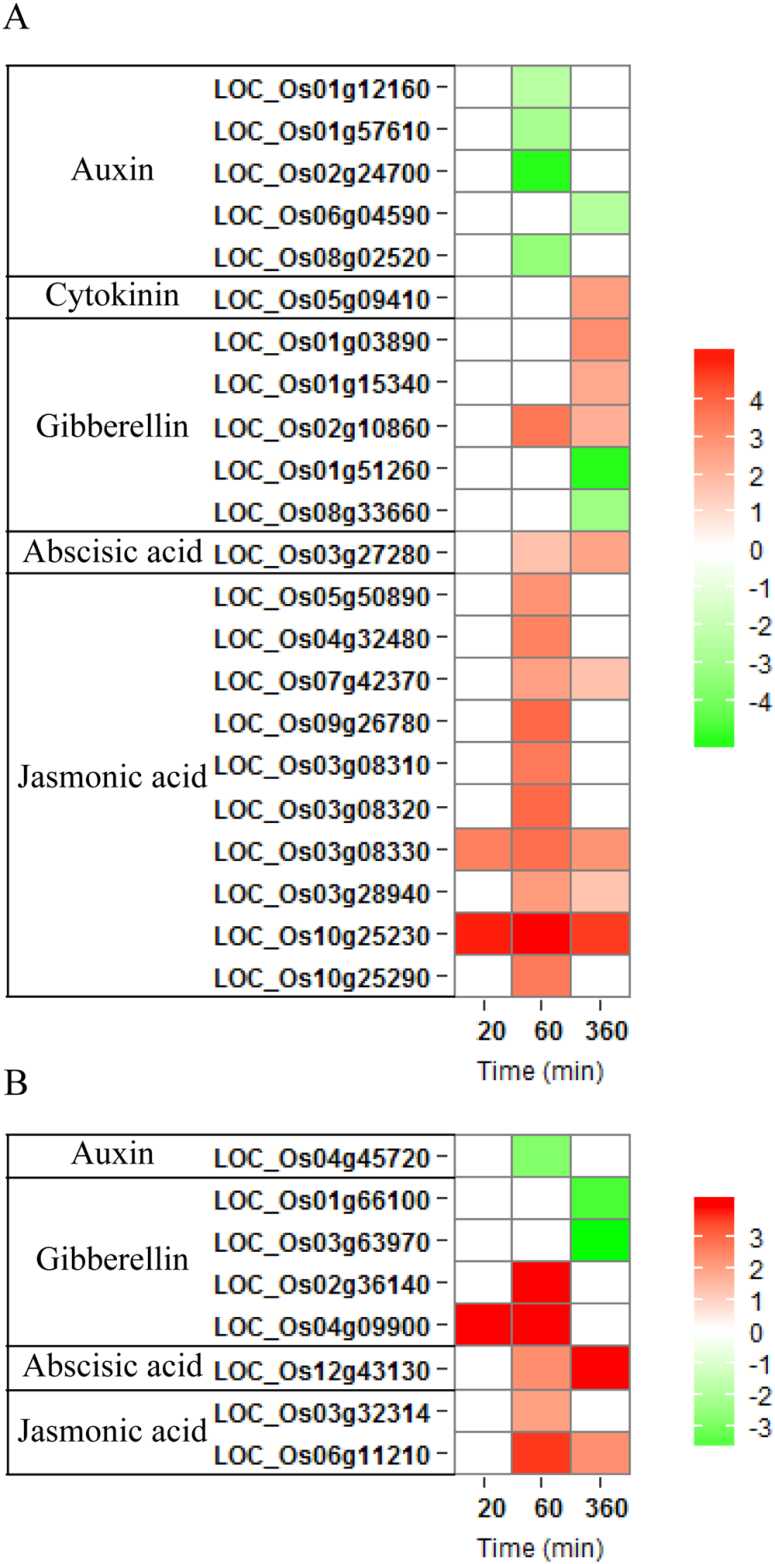
Expression profiles of some focused genes in response to light at three time points. Heat maps illustrating the relative expression profiles of genes related to phytohormone signaling (**A**) and synthesis (**B**), respectively. Red and green colors depict up- and down-regulation relative to control (light/dark), respectively. The scale shows log2 fold change.

The enrichment analysis of GO and KEGG revealed that phytohormone played a central role of light-dependent inhibition of mesocotyl elongation in rice (Table S3 and Fig. 3). We investigated the expression patterns of transcripts associated with signaling or synthesis of hormones, including auxin, cytokinin (CTK), abscisic acid (ABA), gibberellic acid (GA) and jasmonic acid (JA) (Fig. 5A, B). One auxin biosynthesis gene and five auxin signaling genes responded to light, and the genes were all down-regulated after 60 min or 360 min of light exposure. These genes included auxin responsive SAUR gene family member, probable indole-3-acetic acid-amido synthetase (GH3) and aldehyde dehydrogenase (ALDH). In contrast, light enhanced the expression of genes associated with CTK, ABA and JA at one or more time points. The up-regulated genes related to JA had the largest number within the hormone signaling group, including ten JA signaling genes and two JA biosynthesis genes, consisting of seven ZIM domain containing protein genes (JAZ), two zinc-finger protein genes (TF), Jasmonic acid-amino synthetase (JAR1), one 12-oxophytodienoate reductase gene and one allene oxide cyclase gene (AOC). One ABA biosynthesis gene and one ABA signaling gene were up-regulated. Four genes encoding enzymes involved in GA biosynthesis were responsive to light, including two up-regulated genes after 20 min or 60 min of light exposure and two down-regulated genes (*GA20ox1* and *GA20ox2*) after 360 min of light exposure. Of five DEGs associated with GA signaling, three were up-regulated and two were down-regulated.

### Light-triggered phytohormone changes in rice mesocotyl

**Figure 6:**
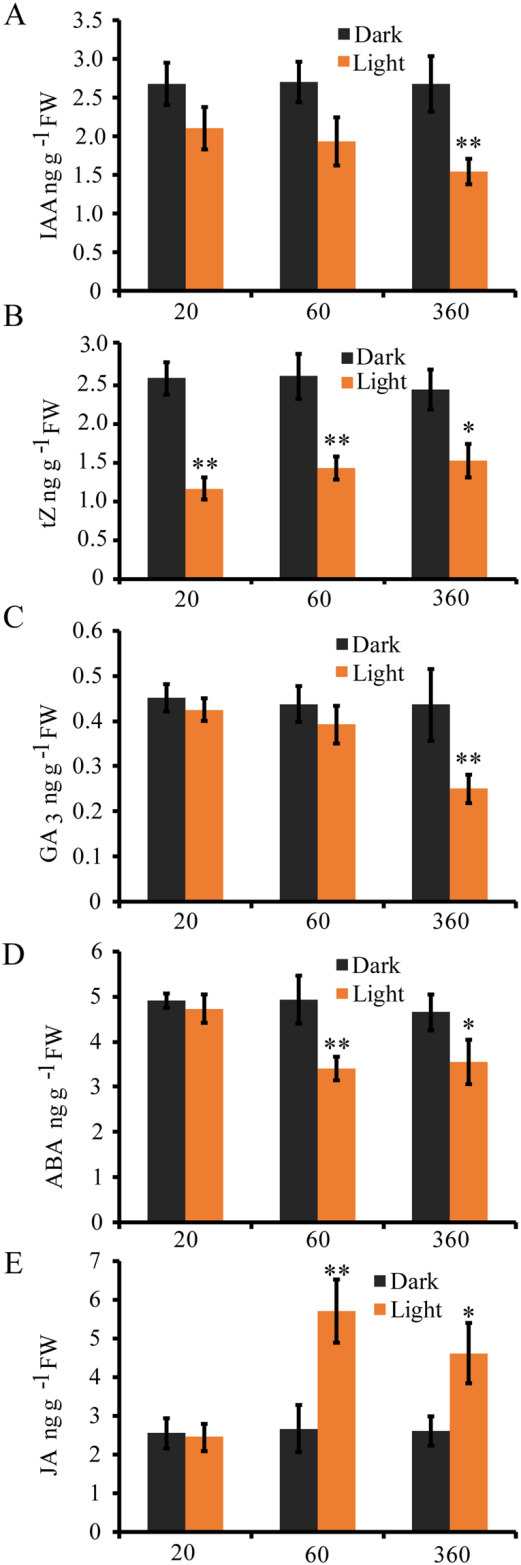
The phytohormone levels in the mesocotyl of the seedlings. The levels of IAA (**A**), tZ (**B**), GA_3_(**C**), ABA (**D**) and JA (**E**) in 3-day-old seedlings raised in complete darkness and irradiated with white light for 20, 60 and 360 minutes. The results represent the average of three biological replicates. The asterisks indicate significant difference compared with control value (**P < 0.01; *P < 0.05, Student’s *t* test).

As phytohormone signaling and biosynthesis gene expression changed after light exposure, we further compared the phytohormone content in mesocotyl between the treatment and the control. After light treatment, the IAA level was gradually decreasing, reaching about 60% of the control at 360 min (Fig. 6A), which was consisting with the down-expression of auxin signaling gene *OsGH3.1* and *OsSAUR24* (Fig. 5A and Table S5). The tZ content decreased to less than one-half of the control at 20 min and slightly increased at the late stage of light treatment (Fig. 6B). The light-induced decrease of IAA and tZ correlates with the reduced growth of mesocotyl. The content of GA_3_ had no significant change at the early stage of light treatment, and changed only at 360 min (Fig. 6C), consistent with the down-expression of GA biosynthesis gene *GA20ox1*and *GA20ox2* at this time point (Fig. 5B and Table S5). Moreover, the ABA level was reduced after 60 min and 360 min of light exposure (Fig. 6D). Among these phytohormones, only JA was significantly increased by 2.1-fold of the control at 60 min and 1.8-fold of the control at 360 min after light treatment (Fig. 6E), in agreement with the up-expression of JA biosynthesis AOC gene (Fig. 5B and Table S5). We also found that exogenous IAA, tZ, GA_3_ and ABA promoted mesocotyl elongation of etiolated rice seedlings, while exogenous JA inhibited mesocotyl elongation of etiolated rice seedlings (Fig. S2). These results indicated that endogenous phytohormone IAA, tZ, GA_3_ and ABA positively regulated rice mesocotyl elongation, while JA negatively modulated the growth of mesocotyl.

## Disscussion

Light is one of the most important environmental factors that impact plant development. The mesocotyl in rice could be highly elongated when seedlings are grown in darkness, whereas mesocotyl elongation is almost completely inhibited after a low fluence of light exposure. For instance, the mesocotyl of rice seedlings showed little growth in darkness for two more days after 1 min pulse of white light at 10 μmol/m^2^ s (Fig. 1C). A similar dosage of red light (60 s, 12 μmol/m^2^ s) caused about 50% inhibition of mesocotyl growth in maize while a fluence of 1 mol/m^2^ decreased the growth rate to about 20% of the dark control^25^. Other experiments observed the quantitative inhibition of mesocotyl elongation of maize seedlings^9,26,27^ or hypocotyl growth of Arabidopsis seedlings^28^. So the mesocotyl growth in rice is more sensitive to light exposure than that in maize or the hypocotyl growth in Arabidopsis.

The coleoptile or mesocotyl of cereal crops and hypocotyl of Arabidopsis or dicotyledonous crops have been widely used as experimental system by many researchers to unravel the physiological and molecular mechanisms underlying the light-repression of plant growth^14,29,30^. In this study, we used RNA-seq approach to analyze the transcriptomic changes in rice mesocotyl along a 20min-60min-360min time-course of white light treatment. Substantial overall changes in the rice mesocotyl transcriptome involved 974 genes, including 629 genes that were significantly induced and 345 genes that were significantly repressed (Table S2). The number of DEGs after 60 min light exposure was 3 or 2 times higher than that of 20 min and 360 min time points, respectively. It is highly noticeable only 20 common genes were detected differentially expressed along three time points (Fig. 2, Table S2). So the light exposure could induce expression changes of a large number of genes in the mesocotyl and quick varied patterns along the time-course of light treatment.

GO and KEGG enrichment analysis showed that these DEGs were mainly associated with transcription factor and plant hormone signaling or biosynthesis. This result could be easily expected as phytohormone was known to play a major role in repression of mesocotyl growth by light^14,25,30^.

Light regulates expression of transcription factors. Transcriptional regulation is one of the major regulatory modes during plant growth and development. Here we found that 77 TF genes belonging to 11 TF families were responsive to light at least at one time point (Fig. 4). The large number of TF genes may reflect the complexity of regulation and a drastic transcriptional reprogramming in rice mesocotyl in response to light. Among the 21-down-regulated TFs, GRF, TCP and GATA could be considered as important candidate genes. In Arabidopsis hypocotyls, TCP4 directly activates YUCCA5 transcription and integrates the auxin response to a brassinosteroid-dependent molecular circuit that promotes cell elongation^31^. AtGRF proteins play a role in the regulation of cell expansion in leaf and cotyledon tissues^32^. GATA gene mutants display a hypocotyl elongation defect in light-grown seedlings^33^. This indicated that the transcription factors of GRF, TCP and GATA play an important role in light-regulation of mesocotyl elongation. Among the 56-up-regulated TF genes, a basic domain/Leucine zipper transcription factor (HY5) was differentially expressed at all three time points (Fig. 4). HY5 was 15 to 20 times more abundant in Arabidopsis seedlings grown in the light than in the dark and involved in the inhibition of hypocotyl elongation by light exposure^24,34^. Plants can perceive light through several kinds of photoreceptors, such as the phytochromes (phys), which absorb red/far-red light^10,11,16^, and the cryptochromes (crys), which absorb blue light^28,35^. The expression of PHY and CRY gene families did not change significantly in this study. There were no significant difference in phytochrome interacting factor (PIL) between light and dark except that *OsPIL14* was slightly up-regulated (Table S6), in agreement with slight increasing of *OsPIL14* transcripts detected by northern blot after exposure to light^29^.

The phytohormones function in light-dependent regulation of mesocotyl elongation. Plants have evolved very efficient cross-talk between light signaling and endogenous phytohormones which have been frequently invoked as effectors of light responses^11,14^. In this study, significant changes in both expression levels of phytohormone-related genes and the contents of phytohormones were detected in response to light (Figs 5, 6 and Table S5). Furthermore, light exposure caused decreased contents of auxin, cytokinin and GA_3_, together with increased JA level. Those changes were compatible with patterns of up- or down-regulations in expression of most auxin- and JA-related genes. The changes of hormone contents and related gene expression levels were not consistent for GA_3_, cytokinin and ABA (Figs 5, 6). These results suggest that light inhibition of mesocotyl elongation could be caused by both lower functioning of growth enhancing phytohormones (IAA, tZ, GA_3_) and higher level of repressing phytohormone (JA).

Auxin regulates multiple plant growth and developmental processes^36^. The early auxin-responsive GH3 genes encode IAA-amido synthetases, which help to maintain auxin homeostasis by conjugating excess IAA to amino acids^37^. The mutant of *OsGH3.1* had low content of free IAA and was insensitive to IAA^38^. We found that two GH3 genes were significantly down-regulated in response to light (Fig. 5, Table S5), which was consistent with the decreased IAA amount in the mesocotyl after exposure to light (Fig. 6A). Three auxin-responsive SAUR genes were also down-regulated (Fig. 5, Table S5), whose homolog (*AtSAUR24*) of Arabidopsis functions in promoting cell expansion and hypocotyls growth^39^. The decrease of IAA level by light might be attributed to the decreased expression of *OsGH3.1* or *OsSAUR24*.

A number of studies have shown that auxin and GA interact to regulate elongation growth in stems and hypocotyls. In Arabidopsis seedlings, auxin regulates the expression of *GA20ox* and *GA3ox* gene families involved in synthesis of active GAs^40^. Consistently, we found that down-regulated expression of *GA20ox1* and *GA20ox2* in rice was later than that of auxin genes (Table S5). The content of GA_3_ in the mesocotyl was also significantly decreased at 360 min of light exposure (Fig. 5C). This suggests that GA_3_ modulates mesocotyl growth at late stage of light induction and may be regulated by auxin.

JA, derived from α-linolenic acid via one branch of the octadecanoid pathway, is an important regulator of coleoptile growth in response to light^14^. JA was substantially and rapidly stimulated by a factor of 10 to 20 in the coleoptile of wild type rice seedling in response to red light. JA was completely absent in *hebiba* mutant that has much longer coleoptile and mesocotyl in darkness or in red light^14^. JA respressed mesocotyl and coleoptiles elongation in etiolated rice seedlings^17^ and inhibited hypocotyls elongation and stimulated cotyledon unfolding in etiolated *Arabidopsis* seedlings^41^. We observed the light-activation of many genes involved in the JA biosynthesis and signaling pathways, such as allene oxide cyclase (AOC), 12-oxophytodienoate reductase, JAR1, JAZ and zinc-figure protein (Table S5). Recent discoveries have shown that JAZ proteins are crucial regulators of the jasmonate hormonal response^42^. We found that all six JAZ genes were up-regulated. This indicates that JA signal transduction may be involved in light-dependent regulation of mesocotyl elongation. The content of JA was significantly increased after 60 min of light exposure, consistent with the up-expression of JA biosynthesis AOC gene (Figs 5B and 6E). JA inhibited mesocotyl elongation of etiolated rice seedlings by exogenous JA application (Fig. S2). All these results indicate that JA may negatively regulate mesocotyl elongation.

## Methods

### Plant materials, growth conditions, and phenotypic measurements

Upland rice landrace ‘Zhaxima’, originally collected from Yunnan, China, have the longest mesocotyl in a collection of rice germplasm^7^. Brown rice grains were sterilized in a 2% sodium hypochlorite solution for 30 min and rinsed three times with sterile water. The sterilized seeds were separately sown on 0.4% (w/v) phytagel in square culture dishes which were stacked in cartons. Then the cartons were covered with black plastic film and placed in a growth chamber with 70% relative humidity (14 h light/10 h dark) for the seeds to germinate and the seedlings to grow at 30 °C under complete darkness. For the time-course experiments, the seedlings in separated cartons were exposed to white light (10 μmol/m^2^s) at each day from the first day to the sixth day after sowing. On the sixth day, mesocotyl lengths were measured using a ruler. Data were presented as the averages and the standard errors of at least 10 seedlings per treatment.

To check the effective period of light interruption, three batches of seedlings were grown in dark for three days, then used for measuring the mesocotyl length at that day (D3), exposed to light for one minute then kept in dark (D3I), and grown in dark until the fifth day (D5), respectively. The mesocotyl length of D3I and D5 seedlings were measured at the fifth day after sowing.

For exogenous phytohormones application experiment, the phytohormones (IAA, tZ, GA_3_, ABA and JA) were dissolved in ethanol, and equivalent volumes of ethanol were added into culture media as the control (CK). Germinated seeds were sown on the culture media (0.4% phytagel) containing 10 µM IAA, 10 µM tZ, 10 µM GA_3_, 1 µM ABA, 1 µM JA respectively. After growth for 2 d at 30 °C in darkness, the lengths of mesocotyls were measured.

### RNA isolation, sequencing and analysis

The dark-grown seedlings were respectively exposed to light for 20 min, 60 min and 360 min on the third day after sowing. At each time point, the mesocotyls of light treated seedlings and control seedlings were collected with three biological replicates and immediately frozen in liquid nitrogen. Total RNA was isolated from a portion of each sample using the PureLink^®^ Plant RNA Reagent (Life Technologies). Another portion of each sample was used to measure the content of endogenous phytohormones.

Sequencing libraries were generated following the specifications of the TruSeq^®^ RNA Sample Preparation Guide V2 (Illumina). Briefly, the mRNAs were enriched using oligo (dT) magnetic beads and broken into short fragments. The cDNA was synthesized using random hexamer primer, M-MuLV Reverse Transcriptase and DNA polymerase I (TaKaRa). The adaptor was ligated to cDNA fragments and purified to select cDNA fragments of approximately 200 bp. PCR amplification was performed with size-selected and adaptor-ligated cDNA with Pfu High-Fidelity DNA Polymerase. PCR products were purified and sequenced with Illumina Hiseq 2000 in Shanghai Majorbio Biopharm Technology Co. Ltd. (Shanghai, China). The raw reads were deposited in the National Center for Biotechnology Information Gene Expression Omnibus (NCBI GEO) with the accession number of PRJNA306542.

The raw reads obtained were pre-processed by removing adaptor sequences and discarding empty reads and low-quality sequences. Then, all clean reads were assembled using the software Cufflinks^43^ and aligned to the reference genome (MUS Rice Genome Annotation Project Release 7, http://rice.plantbiology.msu.edu). The transcript abundance was calculated by estimating FPKM values (fragments per kilobase of transcript sequence per millions base pairs sequenced). The basic information of the transcriptomic data is provided in Table S1. Finally, the Cuffdiff module of Cufflinks^43^ was used to identify differentially expressed genes between light-treated samples and untreated controls. The heat map graph was generated with ggplot2 R package to show gene expression levels.

### Quantitative PCR analysis

RNA was converted to cDNA using PrimeScript RT reagent Kit with gDNA Eraser (TaKaRa, Dalian, China). Real time quantitative PCR (qPCR) was performed in 96-well plate with a Bio-Rad CFX96 Detection System (Bio-Rad) using the SYBR premix EX Taq (TaKaRa). The reaction procedure was as follows: 95 °C for 60 s, followed by 40 cycles at 94 °C for 15 s and 60 °C for 60 s. The rice actin gene (LOC_Os03g50885) was used as the reference gene to normalize the target gene expression, which was calculated using the relative quantization method (2^−ΔΔCT^) as described earlier^44^. For each time point, the gene expression levels were determined by the means of three biological replicates. Specific primer pairs were designed with Primer 5.0 (Table S7).

### Enrichment analyses of gene ontology and KEGG pathways

To analyze the GO enrichment, significantly differential expressed genes at three time points were identified by cutting off based on FDR values < 0.05, and |log2 (fold change)| ≥ 2. GO terms for DEGs were calculated using the goatools python package^45^. KOBAS was employed to analyze the statistical enrichment of DEGs in the Kyoto Encyclopedia of Genes and Genomes (KEGG) pathways^23^. Enrichment analysis of DEGs was performed by using Fisher’s exact test. Significant enrichment was detected with corrected p value < 0.05.

### Extraction and determination of phytohormones

For phytohormone measurement, 300 mg samples were ground to fine power in liquid nitrogen and extracted with 1.5 ml pre-chilled 80% aqueous methanol (v/v) overnight at 4 °C. The supernatant was centrifuged at 13,000 rpm for 20 min at 4 °C, and the solid residue was re-extracted and re-centrifuged. The total supernatant was dried under nitrogen gas and dissolved in 2 ml 0.1 M ammonia solution (v/v). The crude extracts were purified by pre-conditioned Oasis MAX strong anion-exchange column (Waters) and the samples were eluted with 4 ml methanol containing 5% formic acid. The eluent was dried under nitrogen gas and dissolved in 200 µl 80% methanol (v/v) and subjected for ultra-performance liquid chromatography tandem mass spectrometry analysis^46^.

The calibration standards included a mixed phytohormone standard solution containing GA_3_, tZ, ABA, IAA and JA standards (Sigma, USA). The calibration standards were prepared at concentrations of 0.05, 0.1, 0.5, 1, 5, 10 and 20 ng/ml for each phytohormone standard in the mixed standard solution of the five compounds. Calibration standard determinations were repeated four times to develop the standard curve for each compound. The content of each phytohormone was calculated based on the standard curves in the units of ng per g fresh weight (FW). All analyses were performed in three biological replicates.

## Electronic supplementary material

Supplementary tables and figures
