# Supplementary material for: Dynamic transcriptome and phytohormone profiling along the time of light exposure in the mesocotyl of rice seedling
Source: Sci Rep. 2017 Sep 20;7:11961. doi: 10.1038/s41598-017-12326-2 (PMC5607350; doi:10.1038/s41598-017-12326-2)

Supplementary materials

Dynamic transcriptome and phytohormone profiling along the time of light exposure in the mesocotyl of rice seedling

Fangjun Feng, Hanwei Mei, Peiqing Fan, Yanan Li, Xiaoyan Xu, Haibin Wei, Ming Yan *, Lijun Luo *

Table S1. The basic informantion for the transcriptomic data by HisSeq2500.

Table S2. FPKM and fold changes of all DEGs after light treatment. FPKM Ratio conidered significant (q ≤ 0.05, log2|Ratio| ≥ 2) are indicated in red (induced) or green (repressed).

Table S3. Gene ontology (GO) enrichment analysis of DEGs after light treatment.

Table S4. KEGG enrichment analysis of DEGs at 60 minutes after light treatment.

Table S5. Expression profiles of genes related to phytohormone signaling and biosynthesis.

Table S6. Expression profiles of putative phytochrome genes.

Table S7. Primers used for the real-time PCR analysis that was compared with the RNA-Seq analysis.

Figure S1. Comparison of qRT-PCR (blue bar) and RNA-seq (red bar) data for selected gene.

Figure S2. The effects of exogenous phytohormones (IAA, tZ, GA_3, ABA and JA) on mesocotyl elongation of the etiolated rice seedlings grown for 2 d after germination in dark. (A) Phenotype of etiolated seedlings treated respectively with exogenous phytohormones. Arrowheads indicate positions of the coleoptilar nodes between mesocotyl and coleoptile. (B) Mesocotyl length of etiolated seedlings treated respectively with exogenous phytohormones. The values are means ±SD of at least 10 seedlings per treatment. The asterisks indicate significant difference compared with CK (**P < 0.01, Student's *t* test).

Table S1. The basic informantion for the transcriptomic data by HisSeq2500.

Treatment	Total clean reads	Mapped reads	Uniquely mapped	Mapped_rate	Uniq_rate	Number of expressed transcripts (FPKM ≥ 0.2)	Error%	Q20%	Q30%
Dark 20 min rep#1	114699396	109068768	107383970	95.09%	93.62%	28386	0.030	98.04	89.58
Dark 20 min rep#2	120836128	114645588	111462144	94.88%	92.24%	24096	0.030	98.13	89.99
Dark 20 min rep#3	113369084	106966090	105528254	94.35%	93.08%	23601	0.030	98.02	89.53
Light 20 min rep#1	95530310	91085159	89760685	95.35%	93.96%	23035	0.030	98.04	89.58
Light 20 min rep#2	87472336	82769408	81696984	94.62%	93.40%	23931	0.030	98.08	89.82
Light 20 min rep#3	92994180	88569436	87396082	95.24%	93.98%	23720	0.030	98.17	90.12
Dark 60 min rep#1	104589220	99616390	97676632	95.25%	93.39%	24439	0.030	98.10	89.85
Dark 60 min rep#2	93714520	89432258	88041008	95.43%	93.95%	23647	0.030	98.11	89.84
Dark 60 min rep#3	102628842	97527629	96075739	95.03%	93.61%	23553	0.029	98.20	90.27
Light 60 min rep#1	96116458	91517990	90291610	95.22%	93.94%	28373	0.030	98.16	90.07
Light 60 min rep#2	95149590	90692580	89405116	95.32%	93.96%	23496	0.030	98.09	89.83
Light 60 min rep#3	88997834	84652312	83088364	95.12%	93.36%	23727	0.030	98.07	89.69
Dark 360 min rep#1	85667270	80576673	79534629	94.06%	92.84%	22853	0.030	98.09	89.81
Dark 360 min rep#2	97492338	92865905	91328235	95.25%	93.68%	24820	0.030	98.04	89.63
Dark 360 min rep#3	97942082	93136691	91599977	95.09%	93.52%	24300	0.030	98.10	89.83
Light 360 min rep#1	102745708	97477855	95927103	94.87%	93.36%	24252	0.029	98.19	90.28
Light 360 min rep#2	91451208	87042442	85495682	95.18%	93.49%	24210	0.030	98.04	89.59

Light 360 min rep#3	101949156	96702245	95120523	94.85%	93.30%	23568	0.030	98.09	89.79

Table S2. FPKM and fold changes of all DEGs after light treatment. FPKM Ratio considered significant (q ⩽ 0.05, log2|Ratio| ⩾ 2) are indicated in red (induced) or green (repressed).

Genes	20 minutes				60 minutes				360 minutes			
	Dark (FPKM)	Light (FPKM)	Log2(fold_change)	q_value	Dark (FPKM)	Light (FPKM)	Log2(fold_change)	q_value	Dark (FPKM)	Light (FPKM)	Log2(fold_change)	q_value
LOC_Os01g01710					32.371	74.333	1.199	0.0146				
LOC_Os01g01870	5.924	24.400	2.042	0.0230	12.236	72.929	2.575	0.0025				
LOC_Os01g02870					15.356	2.934	-2.388	0.0120	15.475	1.139	-3.764	0.0067
LOC_Os01g02940					51.055	144.428	1.500	0.0078				
LOC_Os01g03330					26.872	178.005	2.728	0.0025	9.137	46.310	2.341	0.0067
LOC_Os01g03490									9.292	2.978	-1.642	0.0444
LOC_Os01g03680	88.956	5.648	-3.977	0.0091								
LOC_Os01g03890									4.075	32.666	3.003	0.0352
LOC_Os01g04260					42.268	13.023	-1.699	0.0133				
LOC_Os01g04330					4.135	22.850	2.466	0.0044				
LOC_Os01g04350					24.583	237.915	3.275	0.0025				
LOC_Os01g04620					15.451	387.588	4.649	0.0025				
LOC_Os01g04800					1.963	23.578	3.586	0.0025	3.008	79.708	4.728	0.0067
LOC_Os01g04920									24.851	6.242	-1.993	0.0152
LOC_Os01g05180					0.141	1.228	3.118	0.0420				
LOC_Os01g05840									18.123	2.186	-3.052	0.0152
LOC_Os01g06210					0.000	0.413	12.012	0.0044				
LOC_Os01g06580									87.876	30.952	-1.505	0.0214
LOC_Os01g06590					11.092	43.607	1.975	0.0025				

LOC_Os01g07090									2.232	13.317	2.577	0.0114
LOC_Os01g07150					10.703	2.083	-2.361	0.0411				
LOC_Os01g07370					31.161	244.823	2.974	0.0025				
LOC_Os01g07530					2.813	25.034	3.154	0.0025	3.519	17.478	2.312	0.0184
LOC_Os01g07570					13.898	4.192	-1.729	0.0182				
LOC_Os01g07880	10.234	91.121	3.154	0.0091	10.568	125.115	3.566	0.0025	17.198	64.935	1.917	0.0152
LOC_Os01g08380					30.898	76.574	1.309	0.0383				
LOC_Os01g08780					6.016	35.261	2.551	0.0025				
LOC_Os01g08890					14.677	54.447	1.891	0.0025				
LOC_Os01g09100	1.109	8.765	2.982	0.0230	3.790	38.141	3.331	0.0025	1.264	11.980	3.244	0.0067
LOC_Os01g09670					12.167	53.965	2.149	0.0025				
LOC_Os01g09770					11.310	49.161	2.120	0.0025				
LOC_Os01g09800					4.583	15.847	1.790	0.0044				
LOC_Os01g10460					9.056	34.525	1.931	0.0078				
LOC_Os01g10850					20.500	0.586	-5.129	0.0105				
LOC_Os01g11230	2.090	31.417	3.910	0.0091	1.178	249.703	7.728	0.0025	6.617	345.205	5.705	0.0067
LOC_Os01g11340					98.165	215.694	1.136	0.0420				
LOC_Os01g11550	3.482	0.089	-5.290	0.0397					12.554	1.556	-3.012	0.0067
LOC_Os01g11710					8.089	0.432	-4.227	0.0025				
LOC_Os01g11730									4.510	94.171	4.384	0.0067
LOC_Os01g11910					12.708	43.996	1.792	0.0025	10.674	42.964	2.009	0.0184
LOC_Os01g12080					8.302	23.363	1.493	0.0092				
LOC_Os01g12160					6.165	1.117	-2.464	0.0061				
LOC_Os01g12420					6.480	1.921	-1.754	0.0383				
LOC_Os01g12570	31.153	3.611	-3.109	0.0091								
LOC_Os01g14410	0.000	3.179	14.956	0.0091	0.000	67.691	19.369	0.0025				
LOC_Os01g14440					8.974	26.800	1.578	0.0216				

LOC_Os01g14550									16.801	4.000	-2.070	0.0376
LOC_Os01g14850	82.723	1.331	-5.958	0.0091					317.145	36.569	-3.117	0.0184
LOC_Os01g15340									5.740	29.291	2.351	0.0067
LOC_Os01g15990					17.166	52.187	1.604	0.0120				
LOC_Os01g16140	21.536	246.174	3.515	0.0091								
LOC_Os01g17000					12.217	3.718	-1.716	0.0293				
LOC_Os01g17380									0.000	1.934	14.239	0.0067
LOC_Os01g17396					1.023	18.793	4.200	0.0025				
LOC_Os01g18170									19.953	89.911	2.172	0.0244
LOC_Os01g19480					11.883	2.315	-2.360	0.0025				
LOC_Os01g19940					2.659	10.410	1.969	0.0326				
LOC_Os01g19970					1.651	13.446	3.026	0.0025				
LOC_Os01g20120									134.818	33.664	-2.002	0.0067
LOC_Os01g20206					2.408	54.218	4.493	0.0025				
LOC_Os01g20950					2.675	0.301	-3.153	0.0205				
LOC_Os01g21034					40.941	4.919	-3.057	0.0025				
LOC_Os01g21120	6.194	310.703	5.649	0.0091								
LOC_Os01g21250					9.514	120.219	3.660	0.0025				
LOC_Os01g21590	7.761	36.793	2.245	0.0448								
LOC_Os01g24030	4.078	195.580	5.584	0.0091					3.223	49.117	3.930	0.0067
LOC_Os01g24550									0.000	1.459	13.833	0.0067
LOC_Os01g24600									0.000	2.205	14.428	0.0067
LOC_Os01g24700					161.023	33.423	-2.268	0.0025	170.219	11.635	-3.871	0.0067
LOC_Os01g25820					45.982	123.966	1.431	0.0061				
LOC_Os01g26039									1.465	11.541	2.978	0.0067
LOC_Os01g27380					0.719	6.682	3.216	0.0061				
LOC_Os01g27390					2.495	24.924	3.321	0.0025	6.011	76.595	3.672	0.0067

LOC_Os01g27480					0.498	3.009	2.595	0.0448				
LOC_Os01g28500					0.000	3.069	14.905	0.0025				
LOC_Os01g29280					0.165	1.463	3.144	0.0227				
LOC_Os01g29330					0.000	0.469	12.196	0.0025				
LOC_Os01g33080					6.253	1.517	-2.044	0.0193				
LOC_Os01g34060	13.481	61.646	2.193	0.0165								
LOC_Os01g34560	7.998	0.249	-5.007	0.0230	5.374	0.193	-4.796	0.0025				
LOC_Os01g35050					80.851	254.023	1.652	0.0025				
LOC_Os01g37350									7.185	1.196	-2.587	0.0067
LOC_Os01g37490					8.324	1.152	-2.854	0.0025				
LOC_Os01g37590					23.857	1.638	-3.864	0.0025	10.440	0.577	-4.176	0.0301
LOC_Os01g39330					5.348	18.672	1.804	0.0025				
LOC_Os01g40630					11.731	32.918	1.489	0.0170				
LOC_Os01g40860					76.525	174.648	1.190	0.0363				
LOC_Os01g41120									31.591	6.218	-2.345	0.0067
LOC_Os01g42234					9.821	31.274	1.671	0.0105				
LOC_Os01g42294									51.974	18.383	-1.499	0.0464
LOC_Os01g42380					15.896	281.668	4.147	0.0025				
LOC_Os01g42410					8.693	52.816	2.603	0.0025				
LOC_Os01g42520					185.444	25.820	-2.844	0.0025				
LOC_Os01g43270					5.059	21.132	2.062	0.0044				
LOC_Os01g43370					33.668	190.807	2.503	0.0025				
LOC_Os01g43372					3.698	19.731	2.415	0.0420				
LOC_Os01g43590					3.338	0.444	-2.912	0.0170				
LOC_Os01g43844					27.123	122.972	2.181	0.0025	31.954	102.735	1.685	0.0324
LOC_Os01g44110					13.790	3.121	-2.144	0.0078	16.318	4.524	-1.851	0.0423
LOC_Os01g45720	1.180	18.788	3.993	0.0091	1.595	63.562	5.317	0.0025	1.877	94.154	5.649	0.0067

LOC_Os01g46970					10.504	40.916	1.962	0.0025				
LOC_Os01g47070					17.186	3.188	-2.430	0.0133				
LOC_Os01g47470					3.688	12.207	1.727	0.0120				
LOC_Os01g48525					0.267	3.355	3.649	0.0479				
LOC_Os01g48530					14.723	91.733	2.639	0.0025				
LOC_Os01g48740									29.231	8.078	-1.855	0.0444
LOC_Os01g49240					0.465	0.000	-12.182	0.0025				
LOC_Os01g49310					4.075	16.876	2.050	0.0025	5.525	43.932	2.991	0.0067
LOC_Os01g50030	12.945	2.199	-2.557	0.0397								
LOC_Os01g50160									4.852	23.086	2.250	0.0067
LOC_Os01g50410	0.908	18.535	4.352	0.0091	1.638	12.284	2.907	0.0273				
LOC_Os01g50420					0.330	1.900	2.525	0.0250				
LOC_Os01g51260									0.464	0.000	-12.181	0.0067
LOC_Os01g51360					26.840	8.479	-1.662	0.0250	32.940	8.208	-2.005	0.0244
LOC_Os01g51570									31.125	247.472	2.991	0.0067
LOC_Os01g51920					35.960	75.808	1.076	0.0485				
LOC_Os01g52180					0.407	0.000	-11.992	0.0025				
LOC_Os01g52260	21.345	148.851	2.802	0.0296	19.947	51.429	1.366	0.0440				
LOC_Os01g52530					19.260	41.886	1.121	0.0326				
LOC_Os01g52640					5.540	21.052	1.926	0.0025				
LOC_Os01g52690					5.703	0.000	-15.799	0.0025				
LOC_Os01g53420					1.586	10.189	2.684	0.0025				
LOC_Os01g53710					40.014	200.079	2.322	0.0025				
LOC_Os01g53730					26.158	77.731	1.571	0.0025				
LOC_Os01g54300									7.371	28.629	1.958	0.0184
LOC_Os01g54340					92.257	33.136	-1.477	0.0105				
LOC_Os01g55100	2.157	0.000	-14.397	0.0091								

LOC_Os01g55150					15.119	0.938	-4.011	0.0025				
LOC_Os01g56180					15.681	37.413	1.255	0.0411				
LOC_Os01g56660					12.764	3.178	-2.006	0.0448	5.335	0.978	-2.448	0.0244
LOC_Os01g56780					0.466	29.736	5.996	0.0025	1.156	24.525	4.407	0.0067
LOC_Os01g56890					43.704	113.406	1.376	0.0105				
LOC_Os01g57030									42.824	7.154	-2.582	0.0376
LOC_Os01g57040					70.326	18.141	-1.955	0.0431				
LOC_Os01g57240					21.414	92.152	2.105	0.0025				
LOC_Os01g57610					1.834	0.241	-2.925	0.0105				
LOC_Os01g58280					0.377	3.705	3.299	0.0025				
LOC_Os01g58420					42.329	106.380	1.330	0.0120				
LOC_Os01g58550					47.096	2.096	-4.490	0.0025	48.977	7.079	-2.791	0.0067
LOC_Os01g58790					11.437	30.468	1.414	0.0078				
LOC_Os01g59150					308.109	125.808	-1.292	0.0394				
LOC_Os01g59680									0.182	9.480	5.699	0.0271
LOC_Os01g59690					7.528	32.739	2.121	0.0025				
LOC_Os01g60020					2.391	11.576	2.275	0.0146				
LOC_Os01g60600					1.492	15.695	3.395	0.0025				
LOC_Os01g60770					311.303	81.607	-1.932	0.0025				
LOC_Os01g60850	0.144	1.963	3.768	0.0091								
LOC_Os01g60940					0.429	7.934	4.208	0.0025				
LOC_Os01g61460					37.026	80.685	1.124	0.0440				
LOC_Os01g61990					9.782	33.369	1.770	0.0025				
LOC_Os01g62110					8.176	0.569	-3.845	0.0193				
LOC_Os01g62430					1.008	9.344	3.213	0.0205				
LOC_Os01g62480									33.359	9.921	-1.750	0.0152
LOC_Os01g62490									77.438	25.425	-1.607	0.0067

LOC_Os01g62810					2.229	15.200	2.770	0.0025				
LOC_Os01g62840					21.472	46.812	1.124	0.0462				
LOC_Os01g62870					13.449	75.166	2.483	0.0025	5.863	34.390	2.552	0.0067
LOC_Os01g63190									111.280	37.068	-1.586	0.0423
LOC_Os01g63210					0.200	98.496	8.943	0.0025				
LOC_Os01g63854					16.825	47.338	1.492	0.0078				
LOC_Os01g64110					21.542	56.858	1.400	0.0061				
LOC_Os01g64180					5.020	0.365	-3.782	0.0092				
LOC_Os01g64250					36.824	15.287	-1.268	0.0440				
LOC_Os01g65370					40.269	151.105	1.908	0.0025				
LOC_Os01g65700									2.056	25.476	3.631	0.0152
LOC_Os01g65980					0.000	0.564	12.462	0.0025				
LOC_Os01g66100									5.403	0.424	-3.670	0.0067
LOC_Os01g67030	0.908	11.677	3.685	0.0165								
LOC_Os01g67480					50.293	150.679	1.583	0.0044				
LOC_Os01g67550									0.872	9.019	3.371	0.0067
LOC_Os01g67810					1.509	8.741	2.534	0.0025				
LOC_Os01g68140					6.016	0.587	-3.357	0.0092				
LOC_Os01g68650					218.354	78.453	-1.477	0.0044				
LOC_Os01g68720									1.442	19.120	3.729	0.0067
LOC_Os01g69290					5.015	0.102	-5.613	0.0061				
LOC_Os01g70470					113.347	272.039	1.263	0.0061				
LOC_Os01g70600					0.274	8.076	4.882	0.0205				
LOC_Os01g70970									7.229	1.671	-2.113	0.0214
LOC_Os01g71170					70.357	8.542	-3.042	0.0025	70.193	10.356	-2.761	0.0067
LOC_Os01g71340					111.862	620.356	2.471	0.0105				
LOC_Os01g71350					11.597	39.077	1.753	0.0092				

LOC_Os01g71420					16.450	4.519	-1.864	0.0078				
LOC_Os01g71790					8.254	27.688	1.746	0.0469				
LOC_Os01g71820					6.961	44.499	2.676	0.0025				
LOC_Os01g71830					7.678	107.541	3.808	0.0025	2.800	58.424	4.383	0.0067
LOC_Os01g71860					0.714	56.869	6.316	0.0025				
LOC_Os01g71970					13.706	40.969	1.580	0.0159				
LOC_Os01g72080					38.980	134.003	1.782	0.0025				
LOC_Os01g72100					43.846	102.860	1.230	0.0469				
LOC_Os01g72520									121.488	42.017	-1.532	0.0324
LOC_Os01g72530	1.089	7.398	2.763	0.0448	1.899	21.688	3.514	0.0025				
LOC_Os01g72700									25.739	9.488	-1.440	0.0214
LOC_Os01g73200					2.550	36.688	3.847	0.0025				
LOC_Os01g73910					7.202	20.280	1.494	0.0455	7.237	28.549	1.980	0.0152
LOC_Os01g74020									17.317	97.613	2.495	0.0067
LOC_Os01g74320					21.833	58.651	1.426	0.0092				
LOC_Os01g74480					5.050	0.859	-2.555	0.0455				
LOC_Os02g01520					9.660	2.525	-1.936	0.0353				
LOC_Os02g01590					103.479	832.154	3.008	0.0025	57.439	194.609	1.761	0.0402
LOC_Os02g02140					4.806	0.212	-4.503	0.0025				
LOC_Os02g02160	0.000	0.506	12.306	0.0091								
LOC_Os02g03060					10.991	30.501	1.473	0.0159				
LOC_Os02g03410					24.354	56.708	1.219	0.0146				
LOC_Os02g03830					12.069	3.176	-1.926	0.0431				
LOC_Os02g03840					29.900	9.403	-1.669	0.0092				
LOC_Os02g04540					19.644	3.083	-2.672	0.0025				
LOC_Os02g04650					22.582	54.041	1.259	0.0261				
LOC_Os02g04810					17.211	4.964	-1.794	0.0025				

LOC_Os02g05470					0.112	2.512	4.482	0.0044				
LOC_Os02g05692					44.466	10.916	-2.026	0.0025				
LOC_Os02g05744					33.642	87.652	1.382	0.0105				
LOC_Os02g07460					8.835	1.167	-2.920	0.0394				
LOC_Os02g07690					22.686	62.973	1.473	0.0078				
LOC_Os02g08150					2.712	37.783	3.800	0.0025	1.095	22.339	4.351	0.0067
LOC_Os02g08220					7.485	1.610	-2.217	0.0353				
LOC_Os02g08270					8.453	71.263	3.076	0.0025				
LOC_Os02g08420					9.270	21.987	1.246	0.0440				
LOC_Os02g08440	1.516	18.022	3.571	0.0091	4.721	27.522	2.544	0.0025				
LOC_Os02g09220					6.410	190.394	4.893	0.0025	4.999	91.104	4.188	0.0067
LOC_Os02g09240					6.082	36.401	2.581	0.0025	3.162	15.641	2.306	0.0152
LOC_Os02g09250									0.000	0.287	11.488	0.0067
LOC_Os02g09930									29.671	183.739	2.631	0.0067
LOC_Os02g10030					0.769	14.628	4.250	0.0044				
LOC_Os02g10500					262.127	114.176	-1.199	0.0455				
LOC_Os02g10520					4.156	18.401	2.147	0.0092	4.961	26.163	2.399	0.0067
LOC_Os02g10550					0.408	9.238	4.502	0.0282				
LOC_Os02g10860					1.606	18.454	3.522	0.0025	3.089	14.129	2.194	0.0444
LOC_Os02g10990					0.147	8.970	5.935	0.0025	0.421	11.019	4.709	0.0214
LOC_Os02g11040	119.970	11.192	-3.422	0.0091	94.667	0.557	-7.408	0.0025				
LOC_Os02g11070					6.864	153.575	4.484	0.0025	2.438	13.216	2.438	0.0184
LOC_Os02g11790	0.959	56.945	5.891	0.0091								
LOC_Os02g11859					7.922	53.034	2.743	0.0025				
LOC_Os02g12270					0.000	2.582	14.656	0.0440				
LOC_Os02g12380	1.152	9.727	3.078	0.0091					1.891	17.008	3.169	0.0067
LOC_Os02g12730									8.310	2.207	-1.913	0.0214

LOC_Os02g13150					18.467	56.363	1.610	0.0025				
LOC_Os02g13660					182.263	26.917	-2.760	0.0025				
LOC_Os02g13710					0.491	2.991	2.606	0.0404	1.121	33.501	4.901	0.0067
LOC_Os02g14160					311.558	117.073	-1.412	0.0044				
LOC_Os02g14910					9.455	0.362	-4.706	0.0216				
LOC_Os02g15640					77.541	21.917	-1.823	0.0025				
LOC_Os02g16030					54.693	128.302	1.230	0.0440				
LOC_Os02g16909					89.085	21.104	-2.078	0.0025				
LOC_Os02g18410					131.614	53.550	-1.297	0.0448				
LOC_Os02g18450					2.358	7.868	1.739	0.0044	2.379	12.135	2.351	0.0114
LOC_Os02g21040					6.545	32.432	2.309	0.0025				
LOC_Os02g21700					22.291	122.911	2.463	0.0025				
LOC_Os02g21920					83.732	202.541	1.274	0.0216				
LOC_Os02g22160					13.830	38.642	1.482	0.0193				
LOC_Os02g22380					77.441	182.550	1.237	0.0159				
LOC_Os02g24700					31.327	0.343	-6.514	0.0025				
LOC_Os02g25230					3.811	12.515	1.715	0.0044				
LOC_Os02g26770					3.346	17.076	2.351	0.0025	3.269	20.578	2.654	0.0324
LOC_Os02g26810					4.911	26.660	2.441	0.0025	5.406	37.502	2.794	0.0067
LOC_Os02g27220					63.934	190.000	1.571	0.0025				
LOC_Os02g30100					11.728	50.552	2.108	0.0025	5.563	37.758	2.763	0.0152
LOC_Os02g32469					577.761	265.168	-1.124	0.0479				
LOC_Os02g32520					10.258	25.760	1.328	0.0146				
LOC_Os02g32690					6.922	22.755	1.717	0.0025	4.491	20.878	2.217	0.0067
LOC_Os02g32970					5.851	18.206	1.638	0.0120				
LOC_Os02g32980					23.181	62.578	1.433	0.0404				
LOC_Os02g33060					34.447	7.296	-2.239	0.0261				

LOC_Os02g33110									4.548	21.022	2.209	0.0114
LOC_Os02g33790					7.459	0.670	-3.476	0.0061				
LOC_Os02g34560					66.235	143.198	1.112	0.0333				
LOC_Os02g35039									19.204	51.151	1.413	0.0464
LOC_Os02g35190					18.165	44.578	1.295	0.0044				
LOC_Os02g35329	0.183	4.710	4.682	0.0230								
LOC_Os02g36030					0.195	13.322	6.092	0.0282				
LOC_Os02g36070	0.000	0.410	12.001	0.0091								
LOC_Os02g36140					0.219	24.122	6.784	0.0025				
LOC_Os02g36190					0.137	13.878	6.666	0.0025				
LOC_Os02g36330					7.270	23.036	1.664	0.0133				
LOC_Os02g36340					32.244	134.868	2.065	0.0025				
LOC_Os02g36530					15.092	134.659	3.157	0.0146				
LOC_Os02g36830					24.341	7.740	-1.653	0.0216				
LOC_Os02g37490	73.940	8.785	-3.073	0.0091	26.993	2.019	-3.741	0.0025	7.655	514.798	6.072	0.0067
LOC_Os02g37690					9.895	42.479	2.102	0.0025	4.951	25.874	2.386	0.0184
LOC_Os02g37780									0.000	1.210	13.563	0.0067
LOC_Os02g37800									23.186	78.890	1.767	0.0184
LOC_Os02g37930					32.827	81.847	1.318	0.0238				
LOC_Os02g38120					75.443	32.425	-1.218	0.0440				
LOC_Os02g38290									75.334	17.528	-2.104	0.0067
LOC_Os02g38320					7.441	36.745	2.304	0.0120				
LOC_Os02g38820					0.749	3.899	2.381	0.0462				
LOC_Os02g39160					20.026	53.925	1.429	0.0078				
LOC_Os02g39330	0.385	4.492	3.546	0.0165								
LOC_Os02g39620	0.000	2.772	14.758	0.0091					0.670	12.597	4.232	0.0184
LOC_Os02g39790					142.399	340.284	1.257	0.0238				

LOC_Os02g40440	0.456	0.000	-12.154	0.0091								
LOC_Os02g40700					3.903	21.246	2.445	0.0061				
LOC_Os02g40840					2.737	21.690	2.986	0.0025				
LOC_Os02g41510					0.387	3.126	3.015	0.0092				
LOC_Os02g41580					15.112	4.274	-1.822	0.0182				
LOC_Os02g41670	3.286	17.009	2.372	0.0230	7.018	59.507	3.084	0.0025				
LOC_Os02g41860									131.200	31.101	-2.077	0.0067
LOC_Os02g41904	109.010	1.450	-6.232	0.0091	75.820	4.802	-3.981	0.0025	69.340	15.229	-2.187	0.0402
LOC_Os02g42380					25.636	2.097	-3.612	0.0044				
LOC_Os02g42660					6.583	0.835	-2.980	0.0326				
LOC_Os02g42850					41.770	103.390	1.308	0.0146				
LOC_Os02g43250					7.649	30.905	2.015	0.0105				
LOC_Os02g43470					0.162	1.336	3.042	0.0133				
LOC_Os02g43620					0.243	1.275	2.388	0.0420				
LOC_Os02g43640									225.530	69.177	-1.705	0.0114
LOC_Os02g43700					22.964	148.447	2.693	0.0025				
LOC_Os02g43790	8.511	52.305	2.620	0.0091	9.735	73.505	2.917	0.0025				
LOC_Os02g43940	5.894	38.565	2.710	0.0345	9.897	60.230	2.605	0.0025				
LOC_Os02g43970					20.084	54.902	1.451	0.0315				
LOC_Os02g44000					18.970	77.407	2.029	0.0025				
LOC_Os02g44010					5.975	19.804	1.729	0.0238				
LOC_Os02g44080									150.084	51.523	-1.543	0.0444
LOC_Os02g44320					39.403	0.198	-7.634	0.0273	221.249	13.428	-4.042	0.0067
LOC_Os02g44820					68.041	18.866	-1.851	0.0061				
LOC_Os02g44980					45.504	112.278	1.303	0.0092				
LOC_Os02g45000					15.037	63.356	2.075	0.0025				
LOC_Os02g45310					5.986	0.516	-3.535	0.0044				

LOC_Os02g45620									7.929	1.561	-2.345	0.0423
LOC_Os02g45710					23.161	144.304	2.639	0.0025				
LOC_Os02g45760					6.847	25.004	1.869	0.0092				
LOC_Os02g45780					19.819	60.760	1.616	0.0105				
LOC_Os02g45870					18.154	67.931	1.904	0.0025				
LOC_Os02g46030									1.478	0.183	-3.013	0.0271
LOC_Os02g46260									19.174	61.834	1.689	0.0152
LOC_Os02g46680	1.926	0.075	-4.673	0.0345								
LOC_Os02g47280	3.175	0.383	-3.051	0.0091	2.731	0.123	-4.469	0.0044				
LOC_Os02g47390									11.433	2.497	-2.195	0.0214
LOC_Os02g47560					3.358	9.347	1.477	0.0216				
LOC_Os02g47580					13.787	41.620	1.594	0.0025				
LOC_Os02g47620									0.434	11.394	4.715	0.0244
LOC_Os02g47860					6.103	14.974	1.295	0.0205				
LOC_Os02g48190					49.018	109.336	1.157	0.0293				
LOC_Os02g48360					14.381	3.138	-2.196	0.0061	16.328	3.963	-2.043	0.0444
LOC_Os02g48730					4.747	1.118	-2.086	0.0420				
LOC_Os02g48770					2.251	14.190	2.656	0.0044				
LOC_Os02g48860					17.116	46.426	1.440	0.0170				
LOC_Os02g50600					80.849	281.820	1.802	0.0025				
LOC_Os02g51070									3.024	17.255	2.513	0.0067
LOC_Os02g51080	1.614	18.015	3.481	0.0165	2.618	31.449	3.587	0.0025	5.173	56.115	3.439	0.0067
LOC_Os02g51320					19.894	0.176	-6.817	0.0025				
LOC_Os02g51730					1.756	25.393	3.854	0.0025				
LOC_Os02g51930					1.978	8.708	2.138	0.0363				
LOC_Os02g52150					16.985	49.809	1.552	0.0105				
LOC_Os02g52210					5.809	24.037	2.049	0.0120				

LOC_Os02g52490					69.826	25.631	-1.446	0.0044				
LOC_Os02g53130									16.461	2.620	-2.651	0.0067
LOC_Os02g53140					5.912	14.417	1.286	0.0193				
LOC_Os02g53200					34.351	11.099	-1.630	0.0333				
LOC_Os02g53690	3.803	0.179	-4.408	0.0091	3.806	0.157	-4.598	0.0105				
LOC_Os02g53720	16.364	0.262	-5.963	0.0091	8.916	0.097	-6.524	0.0394				
LOC_Os02g53835					19.498	69.115	1.826	0.0025				
LOC_Os02g54190									17.480	4.474	-1.966	0.0402
LOC_Os02g54540					13.496	1.145	-3.559	0.0061				
LOC_Os02g54560	5.018	32.180	2.681	0.0091					18.217	2.121	-3.102	0.0214
LOC_Os02g54870					4.811	0.167	-4.844	0.0025				
LOC_Os02g55380					16.380	2.109	-2.957	0.0044				
LOC_Os02g55430					241.421	590.500	1.290	0.0159				
LOC_Os02g55560					8.514	41.070	2.270	0.0025				
LOC_Os02g55970					13.084	47.383	1.857	0.0485				
LOC_Os02g56250					38.873	86.229	1.149	0.0411				
LOC_Os02g56310					56.476	20.057	-1.494	0.0205				
LOC_Os02g56334					1.009	5.597	2.472	0.0025				
LOC_Os02g56370					0.366	3.577	3.289	0.0025				
LOC_Os02g56380					0.203	5.617	4.792	0.0025				
LOC_Os02g56420					0.000	0.193	10.917	0.0025				
LOC_Os02g56700									0.132	142.457	10.070	0.0376
LOC_Os02g56900					46.468	187.412	2.012	0.0025				
LOC_Os02g57110									37.385	2.048	-4.190	0.0114
LOC_Os02g57120					33.962	10.331	-1.717	0.0078				
LOC_Os02g57160					2.937	18.560	2.660	0.0025				
LOC_Os02g58720					20.545	1.928	-3.414	0.0025				

LOC_Os03g01530					31.238	8.149	-1.939	0.0120				
LOC_Os03g01800					37.143	5.131	-2.856	0.0025	42.730	3.113	-3.779	0.0067
LOC_Os03g02240					14.508	3.771	-1.944	0.0092				
LOC_Os03g02450									5.268	26.590	2.336	0.0114
LOC_Os03g02900	0.519	3.554	2.776	0.0397	0.530	8.735	4.043	0.0025				
LOC_Os03g03034	3.340	30.838	3.207	0.0091	13.512	127.081	3.233	0.0025	3.925	42.534	3.438	0.0067
LOC_Os03g03200					15.124	45.953	1.603	0.0250				
LOC_Os03g03450					5.702	28.182	2.305	0.0025				
LOC_Os03g03720					0.114	1.127	3.300	0.0216	1.043	23.444	4.490	0.0464
LOC_Os03g03860					4.173	0.917	-2.187	0.0250				
LOC_Os03g04190									46.255	9.460	-2.290	0.0067
LOC_Os03g04220					20.644	4.478	-2.205	0.0105				
LOC_Os03g04470									10.315	42.267	2.035	0.0324
LOC_Os03g04650					22.185	66.709	1.588	0.0025				
LOC_Os03g04680					46.561	14.604	-1.673	0.0061				
LOC_Os03g04770					8.162	74.044	3.181	0.0025				
LOC_Os03g05110					11.815	31.001	1.392	0.0044				
LOC_Os03g05310					8.273	19.994	1.273	0.0344				
LOC_Os03g05520					39.730	3.930	-3.338	0.0025				
LOC_Os03g05920					5.528	21.531	1.962	0.0025				
LOC_Os03g06090					8.195	27.270	1.734	0.0133				
LOC_Os03g06390					63.708	12.497	-2.350	0.0025				
LOC_Os03g06510					2.773	0.490	-2.501	0.0044				
LOC_Os03g06520					29.119	187.937	2.690	0.0025				
LOC_Os03g06570					12.104	2.819	-2.103	0.0344				
LOC_Os03g06610					34.117	10.952	-1.639	0.0315				
LOC_Os03g06680					53.460	10.607	-2.333	0.0025				

LOC_Os03g06700					3.127	0.069	-5.498	0.0146				
LOC_Os03g06705	9.518	0.323	-4.882	0.0091								
LOC_Os03g06940					7.556	1.929	-1.970	0.0383				
LOC_Os03g07880									14.209	45.688	1.685	0.0352
LOC_Os03g07960					0.720	6.136	3.091	0.0044				
LOC_Os03g08310					1.130	12.457	3.463	0.0044				
LOC_Os03g08320					11.520	165.518	3.845	0.0025				
LOC_Os03g08330	8.195	83.426	3.348	0.0091	13.300	175.761	3.724	0.0025	6.110	44.522	2.865	0.0067
LOC_Os03g08520					0.724	88.822	6.939	0.0025				
LOC_Os03g09220					59.342	16.333	-1.861	0.0025				
LOC_Os03g09250					3.829	12.944	1.757	0.0133				
LOC_Os03g09900					236.542	634.072	1.423	0.0170				
LOC_Os03g10210									0.863	9.042	3.389	0.0152
LOC_Os03g10240					37.057	143.038	1.949	0.0025				
LOC_Os03g10478									184.865	65.948	-1.487	0.0152
LOC_Os03g10640					3.475	9.723	1.485	0.0193				
LOC_Os03g11210					25.777	8.436	-1.612	0.0495				
LOC_Os03g11950					75.186	25.193	-1.577	0.0044				
LOC_Os03g12140					9.495	1.610	-2.560	0.0025	14.590	3.880	-1.911	0.0464
LOC_Os03g12290									48.827	17.617	-1.471	0.0271
LOC_Os03g12390					15.222	69.444	2.190	0.0025				
LOC_Os03g12414					0.360	2.357	2.711	0.0216				
LOC_Os03g12510	39.909	595.846	3.900	0.0165								
LOC_Os03g12820					4.144	14.493	1.806	0.0133				
LOC_Os03g13140	16.895	106.049	2.650	0.0091								
LOC_Os03g13180									0.274	7.264	4.730	0.0067
LOC_Os03g14040					1.877	11.773	2.649	0.0025	2.290	19.537	3.093	0.0114

LOC_Os03g14120					3.204	18.840	2.556	0.0025	3.584	19.027	2.408	0.0214
LOC_Os03g14400					6.828	31.174	2.191	0.0025				
LOC_Os03g14690					7.627	21.227	1.477	0.0092	6.166	49.074	2.993	0.0067
LOC_Os03g14720					4.709	13.446	1.514	0.0061				
LOC_Os03g14730					20.630	88.186	2.096	0.0025	18.777	108.398	2.529	0.0067
LOC_Os03g14840					15.055	1.078	-3.804	0.0025				
LOC_Os03g15230									50.986	12.579	-2.019	0.0067
LOC_Os03g15340									159.321	36.901	-2.110	0.0067
LOC_Os03g16860					91.064	365.867	2.006	0.0025				
LOC_Os03g16960					7.788	25.926	1.735	0.0078	7.237	48.446	2.743	0.0067
LOC_Os03g17000					19.340	48.264	1.319	0.0061				
LOC_Os03g17200	57.016	4.330	-3.719	0.0230								
LOC_Os03g17740					102.657	211.290	1.041	0.0479				
LOC_Os03g17760					22.151	0.112	-7.633	0.0411				
LOC_Os03g17980					24.774	51.662	1.060	0.0293				
LOC_Os03g18030					0.377	13.317	5.141	0.0025				
LOC_Os03g18070					6.639	111.792	4.074	0.0025	5.948	25.365	2.092	0.0114
LOC_Os03g18130					219.102	73.974	-1.567	0.0078	110.137	28.334	-1.959	0.0114
LOC_Os03g18740					1.014	11.491	3.503	0.0044				
LOC_Os03g19200									16.786	6.013	-1.481	0.0444
LOC_Os03g19270	6.633	228.926	5.109	0.0091								
LOC_Os03g19380	0.960	0.000	-13.229	0.0091								
LOC_Os03g19560					0.199	6.403	5.009	0.0146	0.727	7.070	3.283	0.0485
LOC_Os03g19720					16.413	55.849	1.767	0.0411				
LOC_Os03g20090					1.456	12.802	3.137	0.0025				
LOC_Os03g20170					12.194	32.122	1.397	0.0333				
LOC_Os03g20330					1.410	9.436	2.742	0.0344				

LOC_Os03g20380									63.132	19.809	-1.672	0.0114
LOC_Os03g20450									6.351	1.284	-2.307	0.0352
LOC_Os03g20550					33.088	184.449	2.479	0.0025	23.771	90.891	1.935	0.0067
LOC_Os03g20600					18.224	89.471	2.296	0.0025				
LOC_Os03g20949					4.725	15.352	1.700	0.0025				
LOC_Os03g21160	3.834	0.474	-3.015	0.0494	2.803	0.298	-3.231	0.0105				
LOC_Os03g21730					3.079	0.536	-2.523	0.0078				
LOC_Os03g21800					15.706	4.268	-1.880	0.0092				
LOC_Os03g21820					4.955	0.389	-3.672	0.0455				
LOC_Os03g22010									34.287	1.187	-4.852	0.0214
LOC_Os03g22210	3.871	0.000	-15.241	0.0091								
LOC_Os03g22510					3.166	0.513	-2.624	0.0293				
LOC_Os03g23960					12.902	3.246	-1.991	0.0455				
LOC_Os03g24100					0.341	4.167	3.613	0.0146				
LOC_Os03g25270					30.941	69.646	1.171	0.0448				
LOC_Os03g25920					5.779	29.615	2.358	0.0025				
LOC_Os03g26130					1.891	12.515	2.727	0.0495				
LOC_Os03g27080					15.401	43.154	1.487	0.0025				
LOC_Os03g27280					3.284	10.738	1.709	0.0238	2.107	11.763	2.481	0.0244
LOC_Os03g28080					53.728	17.994	-1.578	0.0092				
LOC_Os03g28160	19.372	107.840	2.477	0.0091	21.361	390.723	4.193	0.0025	31.356	449.387	3.841	0.0067
LOC_Os03g28170									3.610	23.180	2.683	0.0067
LOC_Os03g28940					41.944	267.283	2.672	0.0025	29.567	93.941	1.668	0.0067
LOC_Os03g29360					10.320	69.890	2.760	0.0061	16.696	201.203	3.591	0.0067
LOC_Os03g29540					33.764	8.665	-1.962	0.0025				
LOC_Os03g29970					0.255	2.542	3.317	0.0383				
LOC_Os03g30530					3.607	0.454	-2.991	0.0025				

LOC_Os03g31180									1.354	5.653	2.062	0.0423
LOC_Os03g31690					8.044	20.439	1.345	0.0333				
LOC_Os03g32220					0.295	5.906	4.324	0.0193				
LOC_Os03g32230					0.630	11.498	4.190	0.0025				
LOC_Os03g32314					56.277	226.884	2.011	0.0025				
LOC_Os03g32490									3.195	20.889	2.709	0.0464
LOC_Os03g37640					8.880	28.137	1.664	0.0105				
LOC_Os03g38640					3.369	0.475	-2.828	0.0293				
LOC_Os03g40540									13.457	40.448	1.588	0.0423
LOC_Os03g40610					9.929	1.218	-3.027	0.0227				
LOC_Os03g43010					15.872	162.999	3.360	0.0025				
LOC_Os03g45250	0.731	75.080	6.683	0.0091								
LOC_Os03g45420					12.659	35.719	1.497	0.0044				
LOC_Os03g46070					51.856	289.790	2.482	0.0025				
LOC_Os03g47280					2.119	22.462	3.406	0.0025				
LOC_Os03g48540									8.695	26.533	1.610	0.0184
LOC_Os03g48760					0.885	30.477	5.106	0.0120				
LOC_Os03g48770	2.228	43.577	4.290	0.0091	15.826	115.829	2.872	0.0025				
LOC_Os03g48780					7.923	62.783	2.986	0.0025				
LOC_Os03g49350					29.958	202.383	2.756	0.0025				
LOC_Os03g49380					35.752	157.401	2.138	0.0025				
LOC_Os03g49480									6.098	73.428	3.590	0.0067
LOC_Os03g49610					18.588	2.114	-3.136	0.0025	24.555	6.822	-1.848	0.0464
LOC_Os03g49940					4.370	11.756	1.428	0.0448				
LOC_Os03g50870					21.172	51.887	1.293	0.0182				
LOC_Os03g51530	11.087	167.852	3.920	0.0091								
LOC_Os03g51740									0.586	8.686	3.891	0.0067

LOC_Os03g52410	1.706	19.918	3.546	0.0091	1.400	19.057	3.767	0.0025				
LOC_Os03g53010					0.323	8.140	4.656	0.0374				
LOC_Os03g53020	0.436	18.892	5.437	0.0091	2.559	31.043	3.601	0.0025				
LOC_Os03g53150					292.347	106.352	-1.459	0.0374				
LOC_Os03g53340					0.435	2.811	2.691	0.0078				
LOC_Os03g53690	11.586	0.423	-4.774	0.0091								
LOC_Os03g53930					47.320	135.654	1.519	0.0025				
LOC_Os03g54130					91.578	31.576	-1.536	0.0105				
LOC_Os03g54750					84.891	177.117	1.061	0.0404				
LOC_Os03g54760					8.930	28.915	1.695	0.0061				
LOC_Os03g54910									12.852	1.705	-2.914	0.0214
LOC_Os03g55430	5.986	36.247	2.598	0.0296								
LOC_Os03g55540					27.993	174.191	2.638	0.0025				
LOC_Os03g55680	0.000	15.114	17.206	0.0091								
LOC_Os03g55800					31.350	203.036	2.695	0.0025				
LOC_Os03g55820					6.330	18.163	1.521	0.0193				
LOC_Os03g56820					19.895	1.463	-3.765	0.0025				
LOC_Os03g56900					60.653	130.499	1.105	0.0411				
LOC_Os03g57490	0.883	64.923	6.199	0.0091								
LOC_Os03g57640					3.640	25.665	2.818	0.0025				
LOC_Os03g58010					6.449	35.875	2.476	0.0044				
LOC_Os03g58040					45.259	168.245	1.894	0.0025				
LOC_Os03g58230					22.220	8.278	-1.425	0.0353				
LOC_Os03g58350									28.070	7.748	-1.857	0.0352
LOC_Os03g58580					3.447	14.086	2.031	0.0146				
LOC_Os03g58600					4.061	0.262	-3.952	0.0025				
LOC_Os03g58940					27.834	63.013	1.179	0.0485				

LOC_Os03g59040					14.387	3.432	-2.068	0.0205				
LOC_Os03g59100									25.425	5.777	-2.138	0.0114
LOC_Os03g59110									194.907	42.004	-2.214	0.0067
LOC_Os03g59280									36.856	7.352	-2.326	0.0152
LOC_Os03g59300	26.181	2.155	-3.603	0.0345	18.928	0.465	-5.347	0.0025				
LOC_Os03g59320					0.166	1.889	3.509	0.0092				
LOC_Os03g59440									0.000	37.685	18.524	0.0067
LOC_Os03g60080	16.948	98.185	2.534	0.0091	27.207	170.969	2.652	0.0025				
LOC_Os03g60260					60.231	149.923	1.316	0.0293				
LOC_Os03g60740									28.403	102.454	1.851	0.0067
LOC_Os03g61040					5.640	1.407	-2.003	0.0238				
LOC_Os03g61620					12.951	3.566	-1.861	0.0061				
LOC_Os03g61960					98.892	647.562	2.711	0.0025				
LOC_Os03g62230					4.793	16.046	1.743	0.0216				
LOC_Os03g63270					58.226	168.271	1.531	0.0025				
LOC_Os03g63390									103.584	29.745	-1.800	0.0184
LOC_Os03g63500									9.983	44.576	2.159	0.0067
LOC_Os03g63970									32.181	2.021	-3.993	0.0067
LOC_Os03g64260					1.340	7.864	2.553	0.0133				
LOC_Os04g01320									0.688	3.632	2.401	0.0114
LOC_Os04g01354					0.112	14.991	7.066	0.0025				
LOC_Os04g01690					47.135	2.187	-4.430	0.0025				
LOC_Os04g02050					13.671	57.712	2.078	0.0025				
LOC_Os04g02530									1.489	0.122	-3.605	0.0301
LOC_Os04g02910	6.558	22.757	1.795	0.0494	10.016	111.661	3.479	0.0025	8.168	34.704	2.087	0.0067
LOC_Os04g07110					0.102	0.967	3.250	0.0333				
LOC_Os04g09390	40.170	0.864	-5.539	0.0091	8.379	0.355	-4.560	0.0170				

LOC_Os04g09900	0.073	2.959	5.333	0.0165	0.841	26.597	4.983	0.0025				
LOC_Os04g10060					0.372	22.895	5.945	0.0025				
LOC_Os04g10160					0.724	37.109	5.679	0.0025				
LOC_Os04g10350					12.712	85.908	2.757	0.0025				
LOC_Os04g13210					5.746	19.615	1.771	0.0025				
LOC_Os04g16450									503.921	132.086	-1.932	0.0067
LOC_Os04g16722					0.464	3.206	2.788	0.0025				
LOC_Os04g17660	8.334	70.629	3.083	0.0345								
LOC_Os04g20560									0.224	0.000	-11.130	0.0067
LOC_Os04g21160									0.246	0.000	-11.264	0.0067
LOC_Os04g23550					3.992	64.337	4.010	0.0025				
LOC_Os04g23580					4.005	10.393	1.376	0.0455				
LOC_Os04g23890									2.948	10.793	1.872	0.0423
LOC_Os04g24328	8.761	0.966	-3.180	0.0165								
LOC_Os04g24469					8.985	0.698	-3.686	0.0025				
LOC_Os04g25060					1.598	27.939	4.128	0.0025				
LOC_Os04g25440					59.500	196.128	1.721	0.0025	36.359	102.452	1.495	0.0464
LOC_Os04g25650					3.020	48.832	4.015	0.0025				
LOC_Os04g26910					50.065	107.982	1.109	0.0485				
LOC_Os04g29960					0.056	1.638	4.866	0.0479				
LOC_Os04g30490					2.551	8.530	1.742	0.0193				
LOC_Os04g31370									0.000	1.063	13.376	0.0152
LOC_Os04g31520					446.817	186.004	-1.264	0.0485				
LOC_Os04g31924					22.317	56.373	1.337	0.0092				
LOC_Os04g32110					45.886	125.977	1.457	0.0025				
LOC_Os04g32480					0.802	7.799	3.282	0.0092				
LOC_Os04g32540					1.759	0.260	-2.757	0.0363				

LOC_Os04g32790					0.642	0.000	-12.649	0.0025				
LOC_Os04g32980					6.987	22.203	1.668	0.0044				
LOC_Os04g33260					6.701	1.462	-2.196	0.0227				
LOC_Os04g33370	6.838	0.292	-4.551	0.0494								
LOC_Os04g33390					53.302	330.981	2.635	0.0025				
LOC_Os04g33610	0.208	2.889	3.799	0.0230	0.241	7.719	5.000	0.0025				
LOC_Os04g33740									38.860	8.529	-2.188	0.0301
LOC_Os04g34030	0.000	0.461	12.172	0.0091								
LOC_Os04g34600									48.379	360.050	2.896	0.0067
LOC_Os04g35790					24.841	7.261	-1.774	0.0061				
LOC_Os04g36650									0.533	0.000	-12.379	0.0214
LOC_Os04g37430					3.717	20.139	2.438	0.0025				
LOC_Os04g37480									27.356	3.313	-3.046	0.0067
LOC_Os04g37670					4.657	13.585	1.545	0.0092				
LOC_Os04g37770					509.436	139.103	-1.873	0.0025	556.009	117.613	-2.241	0.0067
LOC_Os04g38430									31.978	10.682	-1.582	0.0423
LOC_Os04g38570					10.965	3.097	-1.824	0.0078				
LOC_Os04g38680					2.782	17.405	2.645	0.0025	3.328	29.331	3.140	0.0067
LOC_Os04g39110	21.922	0.950	-4.528	0.0165	21.727	0.366	-5.892	0.0182				
LOC_Os04g39150	44.712	0.745	-5.906	0.0091								
LOC_Os04g39610					13.535	1.079	-3.649	0.0025	12.731	1.373	-3.213	0.0067
LOC_Os04g40600									14.805	61.197	2.047	0.0067
LOC_Os04g40630					23.857	113.209	2.247	0.0025				
LOC_Os04g41280					1.005	0.117	-3.102	0.0333				
LOC_Os04g41460					25.617	54.827	1.098	0.0440				
LOC_Os04g41560									7.189	41.876	2.542	0.0067
LOC_Os04g41620					0.000	2.663	14.701	0.0025				

LOC_Os04g41960					4.281	24.559	2.520	0.0025				
LOC_Os04g41970					67.479	228.068	1.757	0.0025				
LOC_Os04g42020					6.163	26.685	2.114	0.0133				
LOC_Os04g43070					8.349	29.197	1.806	0.0061				
LOC_Os04g43310					15.783	65.565	2.055	0.0044				
LOC_Os04g43680					37.741	98.547	1.385	0.0105				
LOC_Os04g43800					1.003	7.907	2.979	0.0025				
LOC_Os04g43820					5.791	23.975	2.050	0.0238				
LOC_Os04g44420	0.000	6.445	15.976	0.0091								
LOC_Os04g44440					1.333	0.000	-13.703	0.0025				
LOC_Os04g45020					3.672	18.681	2.347	0.0025				
LOC_Os04g45290					20.902	120.200	2.524	0.0025				
LOC_Os04g45330	1.462	0.000	-13.835	0.0091	0.583	0.000	-12.510	0.0025				
LOC_Os04g45490					1.428	12.565	3.137	0.0025	2.180	9.356	2.102	0.0485
LOC_Os04g45720					1.554	0.203	-2.933	0.0159				
LOC_Os04g45730	3.231	27.631	3.096	0.0091	4.754	34.991	2.880	0.0025				
LOC_Os04g45810	5.416	30.236	2.481	0.0091	5.717	41.621	2.864	0.0025				
LOC_Os04g45900					10.491	25.893	1.303	0.0455				
LOC_Os04g46110									89.607	9.604	-3.222	0.0067
LOC_Os04g46220					27.563	73.606	1.417	0.0092				
LOC_Os04g46400	21.487	82.332	1.938	0.0448	22.745	155.036	2.769	0.0025	16.664	65.495	1.975	0.0184
LOC_Os04g46410	0.690	19.468	4.819	0.0091	2.082	46.393	4.478	0.0025				
LOC_Os04g46440	10.303	45.756	2.151	0.0165	13.928	68.373	2.296	0.0025				
LOC_Os04g46740									22.788	1.911	-3.576	0.0244
LOC_Os04g46810	82.594	372.701	2.174	0.0165					227.035	36.393	-2.641	0.0067
LOC_Os04g46980					10.491	27.901	1.411	0.0485				
LOC_Os04g47120					23.636	88.526	1.905	0.0025				

LOC_Os04g47450					4.373	21.599	2.304	0.0105				
LOC_Os04g47780					1.614	17.583	3.446	0.0025	1.152	9.781	3.086	0.0152
LOC_Os04g47810					0.206	1.341	2.701	0.0250				
LOC_Os04g48840					5.433	28.400	2.386	0.0061				
LOC_Os04g49160					13.198	34.298	1.378	0.0383				
LOC_Os04g49260					6.595	1.051	-2.650	0.0353				
LOC_Os04g49350					1.684	5.152	1.613	0.0282				
LOC_Os04g49450									6.520	0.997	-2.709	0.0271
LOC_Os04g49550					5.924	19.911	1.749	0.0383				
LOC_Os04g50940					0.142	2.485	4.128	0.0238				
LOC_Os04g51150					12.373	51.604	2.060	0.0025				
LOC_Os04g51160					1.880	16.308	3.117	0.0025				
LOC_Os04g51190					29.584	7.197	-2.039	0.0044				
LOC_Os04g52090					41.049	136.636	1.735	0.0044				
LOC_Os04g52390					21.781	7.660	-1.508	0.0092				
LOC_Os04g52479					17.635	59.512	1.755	0.0025				
LOC_Os04g52880									103.726	34.481	-1.589	0.0152
LOC_Os04g53190					41.143	150.043	1.867	0.0025				
LOC_Os04g53350					20.785	6.737	-1.625	0.0078				
LOC_Os04g53930					3.384	13.637	2.011	0.0469				
LOC_Os04g53950									37.222	6.160	-2.595	0.0067
LOC_Os04g54220	2.290	110.445	5.592	0.0091								
LOC_Os04g54230	9.345	115.617	3.629	0.0091								
LOC_Os04g54300					299.155	111.131	-1.429	0.0133				
LOC_Os04g54474					124.973	50.142	-1.318	0.0159				
LOC_Os04g54550					0.000	0.759	12.891	0.0025				
LOC_Os04g54560					0.547	6.936	3.663	0.0025				

LOC_Os04g54564					5.375	18.556	1.788	0.0170				
LOC_Os04g55159					87.734	16.601	-2.402	0.0159	135.789	16.296	-3.059	0.0114
LOC_Os04g55220					12.527	38.978	1.638	0.0025	11.765	33.487	1.509	0.0301
LOC_Os04g55230					4.038	19.212	2.250	0.0025				
LOC_Os04g55920					87.639	326.231	1.896	0.0025				
LOC_Os04g56110					2.343	12.992	2.471	0.0061	0.866	14.341	4.049	0.0067
LOC_Os04g56390					185.157	50.378	-1.878	0.0025				
LOC_Os04g56560					0.000	0.997	13.283	0.0025				
LOC_Os04g56700					0.000	0.769	12.909	0.0025				
LOC_Os04g57540					9.825	32.143	1.710	0.0105				
LOC_Os04g57970					63.243	16.828	-1.910	0.0025				
LOC_Os04g58200	10.028	1.047	-3.260	0.0091					38.409	1.114	-5.107	0.0067
LOC_Os04g58810	7.872	33.314	2.081	0.0230	9.211	44.256	2.264	0.0025				
LOC_Os04g58860					83.688	274.829	1.715	0.0025				
LOC_Os04g58890	14.595	59.674	2.032	0.0397	18.942	182.602	3.269	0.0025				
LOC_Os04g59150									22.982	182.631	2.990	0.0067
LOC_Os04g59190									3.227	87.158	4.755	0.0067
LOC_Os04g59420					4.375	0.465	-3.233	0.0193				
LOC_Os04g59440					0.268	12.777	5.577	0.0044				
LOC_Os04g59450					24.372	9.485	-1.362	0.0374				
LOC_Os04g59480					7.100	26.954	1.925	0.0061				
LOC_Os05g01444									0.842	10.379	3.624	0.0114
LOC_Os05g02140					5.076	17.550	1.790	0.0353				
LOC_Os05g02300									70.270	18.185	-1.950	0.0271
LOC_Os05g02310					154.115	404.382	1.392	0.0133				
LOC_Os05g02390					27.837	3.961	-2.813	0.0025				
LOC_Os05g03040					232.805	867.961	1.899	0.0025				

LOC_Os05g03590					18.417	67.560	1.875	0.0025				
LOC_Os05g03610					5.420	19.762	1.866	0.0061	14.150	76.719	2.439	0.0067
LOC_Os05g03620					8.251	26.875	1.704	0.0120				
LOC_Os05g03640					2.256	47.493	4.396	0.0025	0.965	8.459	3.132	0.0184
LOC_Os05g03830					17.259	71.028	2.041	0.0025				
LOC_Os05g04470					12.636	2.692	-2.231	0.0182				
LOC_Os05g04550					5.153	21.234	2.043	0.0025				
LOC_Os05g04610									51.668	17.761	-1.541	0.0485
LOC_Os05g05020					16.887	45.251	1.422	0.0282	10.041	73.920	2.880	0.0067
LOC_Os05g05030					8.977	56.274	2.648	0.0025	4.700	61.699	3.714	0.0067
LOC_Os05g05180					9.951	25.582	1.362	0.0495				
LOC_Os05g05210									8.003	24.967	1.641	0.0184
LOC_Os05g05670					1.943	18.067	3.217	0.0025				
LOC_Os05g05680					1.399	33.739	4.591	0.0025				
LOC_Os05g05940					193.021	431.343	1.160	0.0462				
LOC_Os05g06940					32.181	146.055	2.182	0.0025	31.176	106.794	1.776	0.0324
LOC_Os05g07060									18.891	1.184	-3.995	0.0067
LOC_Os05g07090					8.677	28.665	1.724	0.0078				
LOC_Os05g07810	7.017	56.757	3.016	0.0345								
LOC_Os05g07940					49.270	116.196	1.238	0.0282				
LOC_Os05g09020					10.417	54.311	2.382	0.0025				
LOC_Os05g09410									8.375	52.386	2.645	0.0067
LOC_Os05g09440	10.523	83.364	2.986	0.0091								
LOC_Os05g09480					3.118	11.389	1.869	0.0205				
LOC_Os05g09640					4.792	0.218	-4.455	0.0455				
LOC_Os05g10210	2.986	0.000	-14.866	0.0091								
LOC_Os05g10650	0.928	16.417	4.145	0.0091	2.203	17.377	2.979	0.0025				

LOC_Os05g10670									17.478	3.711	-2.236	0.0152
LOC_Os05g11010					226.202	570.122	1.334	0.0193				
LOC_Os05g12040					0.254	5.636	4.472	0.0105				
LOC_Os05g15530	1.588	23.144	3.865	0.0091					1.670	15.023	3.169	0.0301
LOC_Os05g15880									2.408	17.359	2.850	0.0067
LOC_Os05g19150					17.008	45.535	1.421	0.0159				
LOC_Os05g21180					22.508	51.867	1.204	0.0344				
LOC_Os05g22730									0.334	5.496	4.039	0.0402
LOC_Os05g23740					19.976	54.261	1.442	0.0044				
LOC_Os05g24650					64.572	197.745	1.615	0.0025				
LOC_Os05g25540					9.742	2.111	-2.207	0.0092				
LOC_Os05g25640					159.719	532.805	1.738	0.0025				
LOC_Os05g25650					22.495	114.633	2.349	0.0105				
LOC_Os05g26840					25.127	85.250	1.762	0.0025				
LOC_Os05g27730					33.562	145.496	2.116	0.0025				
LOC_Os05g27780					119.748	22.324	-2.423	0.0025				
LOC_Os05g28740					122.901	22.814	-2.430	0.0025				
LOC_Os05g28870					14.275	91.596	2.682	0.0025				
LOC_Os05g29050					5.882	14.575	1.309	0.0133				
LOC_Os05g30740									81.438	21.797	-1.902	0.0067
LOC_Os05g30760					0.552	3.138	2.506	0.0404				
LOC_Os05g31280									23.731	1.188	-4.321	0.0324
LOC_Os05g31740					0.710	4.283	2.594	0.0182				
LOC_Os05g32270					14.160	43.721	1.627	0.0092				
LOC_Os05g32970					33.359	74.423	1.158	0.0485				
LOC_Os05g33310					35.614	94.725	1.411	0.0025				
LOC_Os05g33320					37.644	107.699	1.517	0.0170				

LOC_Os05g33400					87.294	360.295	2.045	0.0025				
LOC_Os05g33410									0.278	6.736	4.600	0.0067
LOC_Os05g33730					32.081	90.403	1.495	0.0044				
LOC_Os05g34150	11.596	0.327	-5.147	0.0345	12.267	0.466	-4.717	0.0146				
LOC_Os05g34170									120.089	34.256	-1.810	0.0114
LOC_Os05g34980	9.097	36.858	2.019	0.0448								
LOC_Os05g35010					25.178	6.157	-2.032	0.0044	22.168	5.957	-1.896	0.0114
LOC_Os05g35070					1.569	0.057	-4.786	0.0159				
LOC_Os05g35290					31.954	321.276	3.330	0.0025	15.940	60.231	1.918	0.0067
LOC_Os05g35340									4.102	18.307	2.158	0.0376
LOC_Os05g35380					2.947	0.116	-4.670	0.0092				
LOC_Os05g35410					5.609	18.808	1.745	0.0044				
LOC_Os05g36010					28.066	7.526	-1.899	0.0044	26.906	7.050	-1.932	0.0114
LOC_Os05g36280									103.774	31.662	-1.713	0.0376
LOC_Os05g37250					1.817	11.110	2.612	0.0044				
LOC_Os05g37780	0.182	10.289	5.823	0.0091								
LOC_Os05g37820					23.866	78.379	1.716	0.0025				
LOC_Os05g37900					17.379	4.884	-1.831	0.0146				
LOC_Os05g38140					73.252	211.744	1.531	0.0025				
LOC_Os05g38264	11.420	88.393	2.952	0.0091								
LOC_Os05g38270					2.324	12.716	2.452	0.0025				
LOC_Os05g38390									39.939	12.511	-1.675	0.0184
LOC_Os05g38420									6.017	0.544	-3.469	0.0067
LOC_Os05g38660					29.957	4.262	-2.813	0.0025				
LOC_Os05g39240					7.444	29.383	1.981	0.0061				
LOC_Os05g39310	0.199	76.278	8.582	0.0397					0.193	6.897	5.156	0.0301
LOC_Os05g39350					23.820	56.247	1.240	0.0462	15.093	54.484	1.852	0.0152

LOC_Os05g39990					110.880	6.370	-4.122	0.0025				
LOC_Os05g41780					109.356	385.641	1.818	0.0025				
LOC_Os05g41990					160.273	30.729	-2.383	0.0025	111.651	8.811	-3.664	0.0067
LOC_Os05g42060					2.937	15.902	2.437	0.0092				
LOC_Os05g42070					2.052	44.336	4.433	0.0025				
LOC_Os05g42080					3.197	75.926	4.570	0.0420				
LOC_Os05g42250									15.663	3.544	-2.144	0.0271
LOC_Os05g43170									6.380	28.757	2.172	0.0067
LOC_Os05g43510					1.626	12.966	2.996	0.0205				
LOC_Os05g43690					46.621	10.313	-2.177	0.0044				
LOC_Os05g44060					22.099	89.716	2.021	0.0025				
LOC_Os05g44200					18.604	0.371	-5.647	0.0078				
LOC_Os05g44600									24.233	4.618	-2.392	0.0152
LOC_Os05g44770					15.334	47.280	1.625	0.0061				
LOC_Os05g45020					66.719	13.020	-2.357	0.0025				
LOC_Os05g45410					2.292	6.948	1.600	0.0469				
LOC_Os05g45430					19.433	2.062	-3.237	0.0025				
LOC_Os05g45820					2.136	0.160	-3.735	0.0061				
LOC_Os05g46020	2.270	19.797	3.125	0.0091	3.455	42.889	3.634	0.0025				
LOC_Os05g46240					36.042	101.640	1.496	0.0170				
LOC_Os05g48040					50.528	14.846	-1.767	0.0061				
LOC_Os05g48160									0.244	8.333	5.094	0.0271
LOC_Os05g48200	0.114	13.780	6.915	0.0091								
LOC_Os05g48260					4.026	13.517	1.747	0.0159				
LOC_Os05g48760					14.061	31.591	1.168	0.0305				
LOC_Os05g48810					15.915	44.273	1.476	0.0061				
LOC_Os05g48930					19.707	91.037	2.208	0.0025				

LOC_Os05g49140					70.728	251.186	1.828	0.0025				
LOC_Os05g49420					28.826	106.478	1.885	0.0025				
LOC_Os05g49590					8.019	18.203	1.183	0.0315				
LOC_Os05g49750					4.744	11.046	1.219	0.0411				
LOC_Os05g50100					13.340	69.780	2.387	0.0025				
LOC_Os05g50260					33.696	209.026	2.633	0.0025				
LOC_Os05g50300					0.126	1.624	3.684	0.0146	0.158	5.049	4.995	0.0423
LOC_Os05g50310					4.976	17.542	1.818	0.0193	3.909	19.578	2.325	0.0402
LOC_Os05g50380					14.212	2.661	-2.417	0.0044				
LOC_Os05g50500					20.288	4.556	-2.155	0.0216	29.225	5.094	-2.520	0.0067
LOC_Os05g50550					10.186	30.940	1.603	0.0025				
LOC_Os05g50610					0.059	1.063	4.161	0.0333				
LOC_Os05g50800					15.499	4.950	-1.647	0.0193				
LOC_Os05g50890					20.623	154.277	2.903	0.0025				
LOC_Os05g50960									3.673	0.549	-2.743	0.0301
LOC_Os05g50990					24.518	6.494	-1.917	0.0025				
LOC_Os05g51220					3.234	0.455	-2.830	0.0133				
LOC_Os05g51780					12.933	2.989	-2.113	0.0353				
LOC_Os06g02900									16.524	1.802	-3.197	0.0184
LOC_Os06g03520	7.074	124.122	4.133	0.0091								
LOC_Os06g03560									1.012	4.364	2.108	0.0485
LOC_Os06g03640					163.300	568.293	1.799	0.0025				
LOC_Os06g03810					7.914	36.610	2.210	0.0025				
LOC_Os06g03970	8.821	0.274	-5.007	0.0091	3.270	0.123	-4.730	0.0025				
LOC_Os06g04020					274.846	119.416	-1.203	0.0326				
LOC_Os06g04590									23.502	3.909	-2.588	0.0423
LOC_Os06g04850					21.667	0.598	-5.179	0.0025	18.031	0.872	-4.370	0.0184

LOC_Os06g04870					32.679	12.070	-1.437	0.0374				
LOC_Os06g04940	4.452	224.228	5.655	0.0091								
LOC_Os06g04950	5.081	180.343	5.149	0.0091								
LOC_Os06g05000	36.914	700.178	4.246	0.0091								
LOC_Os06g05020	32.472	442.248	3.768	0.0345								
LOC_Os06g05359					3.832	1.221	-1.650	0.0363				
LOC_Os06g05430									18.132	66.691	1.879	0.0376
LOC_Os06g05440					48.967	16.249	-1.591	0.0293				
LOC_Os06g05550					12.974	1.255	-3.370	0.0025				
LOC_Os06g06180	0.390	15.559	5.317	0.0091	0.404	96.488	7.899	0.0025	0.820	38.596	5.556	0.0067
LOC_Os06g06190					3.113	8.719	1.486	0.0333				
LOC_Os06g06230					0.000	0.448	12.131	0.0044				
LOC_Os06g06250									360.823	115.463	-1.644	0.0214
LOC_Os06g06860					1.144	0.160	-2.834	0.0315				
LOC_Os06g07914									1.488	41.353	4.797	0.0067
LOC_Os06g07932					0.316	2.609	3.046	0.0216				
LOC_Os06g08014									0.976	24.952	4.676	0.0485
LOC_Os06g08032					0.268	2.357	3.139	0.0092				
LOC_Os06g08610									2.020	13.683	2.760	0.0444
LOC_Os06g08640					39.135	97.576	1.318	0.0146				
LOC_Os06g09310					15.110	66.696	2.142	0.0025				
LOC_Os06g09340					5.258	0.628	-3.067	0.0105				
LOC_Os06g10100					1.656	13.554	3.033	0.0061	0.753	6.927	3.201	0.0184
LOC_Os06g10210					6.553	17.040	1.379	0.0216				
LOC_Os06g10230	11.944	0.402	-4.894	0.0091	7.329	0.062	-6.883	0.0025				
LOC_Os06g10310	2.223	0.000	-14.440	0.0091	1.584	0.000	-13.951	0.0025				
LOC_Os06g10880					15.043	57.649	1.938	0.0025				

LOC_Os06g10980					0.709	5.305	2.904	0.0133				
LOC_Os06g11150					7.345	45.263	2.624	0.0025				
LOC_Os06g11210					36.760	470.537	3.678	0.0025	16.028	80.688	2.332	0.0067
LOC_Os06g11240					57.695	243.600	2.078	0.0025				
LOC_Os06g11290					7.489	20.731	1.469	0.0238				
LOC_Os06g11310									100.997	39.932	-1.339	0.0376
LOC_Os06g11450					3.497	13.428	1.941	0.0078				
LOC_Os06g11760					21.283	167.090	2.973	0.0025	19.200	95.674	2.317	0.0352
LOC_Os06g13180	0.612	13.064	4.415	0.0091	1.547	21.312	3.784	0.0025				
LOC_Os06g13190					6.705	96.580	3.848	0.0025	4.974	23.193	2.221	0.0444
LOC_Os06g13280									0.445	0.000	-12.121	0.0067
LOC_Os06g13320					0.263	6.910	4.717	0.0025				
LOC_Os06g14180									0.492	17.552	5.156	0.0067
LOC_Os06g14400									7.319	30.336	2.051	0.0214
LOC_Os06g14490					18.058	45.721	1.340	0.0133				
LOC_Os06g14540					5.401	0.244	-4.470	0.0305				
LOC_Os06g15620					83.652	4.366	-4.260	0.0025				
LOC_Os06g16240									5.653	27.502	2.282	0.0114
LOC_Os06g17000	8.207	0.555	-3.886	0.0091								
LOC_Os06g19070					1.738	12.908	2.893	0.0025	1.337	14.198	3.409	0.0152
LOC_Os06g19370					12.615	30.144	1.257	0.0092				
LOC_Os06g19800									39.750	8.634	-2.203	0.0067
LOC_Os06g20200					9.954	1.468	-2.761	0.0025				
LOC_Os06g21380					0.247	8.302	5.069	0.0025				
LOC_Os06g23720					0.168	0.000	-10.713	0.0120				
LOC_Os06g23910									0.270	0.000	-11.397	0.0067
LOC_Os06g24404									57.192	16.326	-1.809	0.0114

LOC_Os06g24730					24.468	55.096	1.171	0.0333				
LOC_Os06g29220					0.516	7.152	3.793	0.0025				
LOC_Os06g30640					3.696	29.293	2.986	0.0025				
LOC_Os06g30970					51.352	149.111	1.538	0.0025				
LOC_Os06g31800					4391.613	1303.284	-1.753	0.0282				
LOC_Os06g31930					2632.147	865.104	-1.605	0.0333				
LOC_Os06g31960					844.354	296.273	-1.511	0.0227				
LOC_Os06g32020					261.096	94.103	-1.472	0.0159				
LOC_Os06g32160					320.464	97.562	-1.716	0.0025				
LOC_Os06g32240					617.025	181.821	-1.763	0.0193				
LOC_Os06g32350	410.972	28.703	-3.840	0.0091	210.641	13.343	-3.981	0.0025				
LOC_Os06g32355	278.296	18.038	-3.948	0.0091	109.086	9.583	-3.509	0.0092				
LOC_Os06g32370	256.161	22.688	-3.497	0.0091	137.640	9.532	-3.852	0.0025				
LOC_Os06g34120					93.069	26.218	-1.828	0.0044				
LOC_Os06g34650					31.436	8.850	-1.829	0.0333				
LOC_Os06g35410					22.414	51.846	1.210	0.0170				
LOC_Os06g35480					1.493	8.672	2.538	0.0315				
LOC_Os06g35560	4.900	26.352	2.427	0.0091								
LOC_Os06g35650					0.294	4.410	3.906	0.0025				
LOC_Os06g35970					43.751	13.058	-1.744	0.0250				
LOC_Os06g36000									0.939	9.679	3.366	0.0244
LOC_Os06g36040					0.529	6.098	3.526	0.0133				
LOC_Os06g36180					4.612	13.472	1.547	0.0227				
LOC_Os06g36330					3.946	0.373	-3.405	0.0061				
LOC_Os06g36520					0.194	0.000	-10.923	0.0044				
LOC_Os06g37300					0.215	20.100	6.548	0.0025				
LOC_Os06g37610					0.685	10.788	3.978	0.0025	4.280	54.786	3.678	0.0067

LOC_Os06g37620					4.152	12.306	1.568	0.0105				
LOC_Os06g38320									3.346	32.462	3.278	0.0067
LOC_Os06g38660					6.507	26.235	2.011	0.0282				
LOC_Os06g39040					1.893	21.467	3.503	0.0025	1.827	47.529	4.701	0.0067
LOC_Os06g39230					7.842	1.481	-2.405	0.0133				
LOC_Os06g39240	15.101	214.916	3.831	0.0091								
LOC_Os06g39270					5.786	21.306	1.881	0.0261				
LOC_Os06g39370	3.271	57.923	4.146	0.0091								
LOC_Os06g39520					29.342	72.875	1.313	0.0044				
LOC_Os06g40170					5.374	30.578	2.509	0.0025				
LOC_Os06g40180					4.963	21.569	2.120	0.0025				
LOC_Os06g41020									9.071	2.389	-1.925	0.0067
LOC_Os06g41060					5.707	0.454	-3.651	0.0044				
LOC_Os06g41360					14.177	49.010	1.790	0.0025				
LOC_Os06g41860					5.053	0.799	-2.660	0.0159				
LOC_Os06g43090					23.201	84.280	1.861	0.0025				
LOC_Os06g43170					21.214	5.231	-2.020	0.0025				
LOC_Os06g43600					67.498	3.954	-4.094	0.0025				
LOC_Os06g43800									3.471	20.760	2.581	0.0067
LOC_Os06g44010					1.314	11.514	3.132	0.0025				
LOC_Os06g44300					2.968	0.760	-1.965	0.0282				
LOC_Os06g44320					9.957	0.503	-4.306	0.0025				
LOC_Os06g44410					12.915	4.417	-1.548	0.0193				
LOC_Os06g45060	28.346	153.715	2.439	0.0091								
LOC_Os06g45100					0.507	4.245	3.065	0.0105	0.281	7.321	4.704	0.0067
LOC_Os06g45140					12.262	31.819	1.376	0.0462				
LOC_Os06g45570					15.175	59.816	1.979	0.0092				

LOC_Os06g45820					16.820	137.851	3.035	0.0025				
LOC_Os06g46500					13.792	3.635	-1.924	0.0374				
LOC_Os06g46680					11.322	2.140	-2.403	0.0025				
LOC_Os06g46950					18.863	95.367	2.338	0.0025				
LOC_Os06g47600	40.943	3.965	-3.368	0.0091	50.660	1.167	-5.440	0.0025				
LOC_Os06g47620					15.936	83.278	2.386	0.0025				
LOC_Os06g47910	4.490	0.721	-2.639	0.0494								
LOC_Os06g48065					9.943	2.133	-2.221	0.0146				
LOC_Os06g48160					97.768	39.068	-1.323	0.0469				
LOC_Os06g48500					97.402	26.868	-1.858	0.0025				
LOC_Os06g49030					25.008	56.092	1.165	0.0344				
LOC_Os06g49190	712.827	1.574	-8.823	0.0091	69.313	1.850	-5.227	0.0025				
LOC_Os06g49340									0.000	1.148	13.487	0.0067
LOC_Os06g49770					76.437	14.485	-2.400	0.0025	98.278	13.629	-2.850	0.0067
LOC_Os06g50080					177.841	42.471	-2.066	0.0025				
LOC_Os06g50950					4.096	86.638	4.403	0.0025	1.299	21.212	4.030	0.0067
LOC_Os06g51029					8.434	61.457	2.865	0.0025	10.712	40.165	1.907	0.0114
LOC_Os06g51260									14.154	3.946	-1.843	0.0464
LOC_Os06g51270					8.082	2.038	-1.988	0.0193				
LOC_Os06g51360					9.950	43.153	2.117	0.0025				
LOC_Os07g01020					44.921	117.100	1.382	0.0170	22.792	74.869	1.716	0.0214
LOC_Os07g01370									92.476	32.595	-1.504	0.0301
LOC_Os07g01410	5.113	41.079	3.006	0.0165								
LOC_Os07g01440									15.635	0.417	-5.230	0.0402
LOC_Os07g01860					6.249	1.023	-2.611	0.0025				
LOC_Os07g02460					13.214	99.397	2.911	0.0025				
LOC_Os07g03120	2.501	58.662	4.552	0.0091	4.628	209.133	5.498	0.0025	3.429	67.433	4.297	0.0067

LOC_Os07g03170					7.226	41.637	2.527	0.0061				
LOC_Os07g03368					524.456	1473.323	1.490	0.0353				
LOC_Os07g03580					0.000	2.083	14.347	0.0025				
LOC_Os07g03710					189.115	501.466	1.407	0.0146				
LOC_Os07g04560					0.425	6.369	3.906	0.0025				
LOC_Os07g05190									6.659	1.204	-2.468	0.0271
LOC_Os07g05370					12.902	3.205	-2.009	0.0078				
LOC_Os07g05570					13.416	43.571	1.699	0.0025	12.433	106.074	3.093	0.0067
LOC_Os07g06620	2.554	0.126	-4.339	0.0091								
LOC_Os07g06680					185.468	500.716	1.433	0.0120				
LOC_Os07g07320					24.932	79.608	1.675	0.0025				
LOC_Os07g07950					7.142	2.226	-1.682	0.0469				
LOC_Os07g08150					0.000	41.521	18.664	0.0025				
LOC_Os07g08160	0.000	1.476	13.849	0.0091	0.000	25.497	17.960	0.0025	0.000	103.017	19.974	0.0067
LOC_Os07g08390					72.591	25.085	-1.533	0.0078				
LOC_Os07g09190					1.880	20.514	3.448	0.0025				
LOC_Os07g09420					13.701	90.635	2.726	0.0025	7.357	55.410	2.913	0.0067
LOC_Os07g09630	1.416	16.709	3.561	0.0091								
LOC_Os07g10630					5.873	1.914	-1.617	0.0205				
LOC_Os07g11739					0.000	8.926	16.446	0.0025				
LOC_Os07g11790					0.000	0.205	11.005	0.0025				
LOC_Os07g11870					0.000	2.609	14.671	0.0025				
LOC_Os07g13634					1.368	5.334	1.963	0.0282				
LOC_Os07g14850					145.991	358.641	1.297	0.0182				
LOC_Os07g17250					1.636	4.227	1.370	0.0448				
LOC_Os07g17330					40.106	133.756	1.738	0.0044				
LOC_Os07g18120					29.044	77.534	1.417	0.0044				

LOC_Os07g23120	23.629	3.077	-2.941	0.0091								
LOC_Os07g23430					2.201	15.787	2.842	0.0061				
LOC_Os07g27780					10.142	51.829	2.353	0.0025				
LOC_Os07g28400									7.366	40.910	2.474	0.0271
LOC_Os07g30760	2.826	22.561	2.997	0.0091								
LOC_Os07g30960					0.147	5.097	5.113	0.0061				
LOC_Os07g32340					33.115	12.962	-1.353	0.0462				
LOC_Os07g32570					83.148	235.941	1.505	0.0092				
LOC_Os07g32630					2.590	25.055	3.274	0.0025	4.642	29.067	2.647	0.0067
LOC_Os07g32790					13.330	4.029	-1.726	0.0238				
LOC_Os07g33270					17.946	47.771	1.413	0.0315				
LOC_Os07g33660									6.526	44.959	2.784	0.0067
LOC_Os07g33780					13.020	38.191	1.553	0.0146				
LOC_Os07g34260					17.150	94.130	2.457	0.0044				
LOC_Os07g35310	7.350	25.367	1.787	0.0494								
LOC_Os07g35380									0.483	2.927	2.599	0.0271
LOC_Os07g35560									2.750	11.168	2.022	0.0485
LOC_Os07g35740	0.279	6.616	4.565	0.0091								
LOC_Os07g36610					1.576	0.204	-2.953	0.0146				
LOC_Os07g37180					9.092	21.295	1.228	0.0282				
LOC_Os07g37230					0.706	6.454	3.193	0.0044				
LOC_Os07g37320					3.518	16.360	2.217	0.0159				
LOC_Os07g37350					8.198	1.525	-2.426	0.0092				
LOC_Os07g37454	0.125	11.515	6.528	0.0494								
LOC_Os07g37730					0.299	14.676	5.615	0.0061				
LOC_Os07g37810					15.906	4.223	-1.913	0.0025				
LOC_Os07g37850									86.235	5.210	-4.049	0.0324

LOC_Os07g38130					330.284	18.400	-4.166	0.0025				
LOC_Os07g38370					10.914	46.652	2.096	0.0025				
LOC_Os07g38440					1.215	13.933	3.520	0.0025	1.689	38.149	4.497	0.0067
LOC_Os07g38590					39.660	13.781	-1.525	0.0044				
LOC_Os07g38730					436.079	175.561	-1.313	0.0485				
LOC_Os07g39270					16.414	60.460	1.881	0.0025				
LOC_Os07g39860	1.205	12.424	3.366	0.0091	1.911	30.394	3.991	0.0025				
LOC_Os07g40630					11.005	51.293	2.221	0.0025				
LOC_Os07g41200					10.746	3.322	-1.694	0.0092	16.357	4.450	-1.878	0.0152
LOC_Os07g41600					144.394	321.721	1.156	0.0363				
LOC_Os07g42370					190.246	1124.540	2.563	0.0025	155.051	512.013	1.723	0.0152
LOC_Os07g42510									32.365	105.312	1.702	0.0244
LOC_Os07g42740					6.061	20.308	1.744	0.0344				
LOC_Os07g42940					42.124	90.696	1.106	0.0455				
LOC_Os07g42960					199.116	583.602	1.551	0.0092				
LOC_Os07g43240									0.365	3.091	3.080	0.0244
LOC_Os07g43740	5.349	47.235	3.143	0.0091	9.555	43.171	2.176	0.0025				
LOC_Os07g43800					17.248	68.667	1.993	0.0044				
LOC_Os07g43925					6.461	22.958	1.829	0.0216	7.416	32.032	2.111	0.0152
LOC_Os07g43970	2.246	12.777	2.508	0.0296	2.717	7.247	1.416	0.0315				
LOC_Os07g44090					18.527	50.504	1.447	0.0170				
LOC_Os07g44290					7.087	21.623	1.609	0.0025				
LOC_Os07g44690					2.190	0.186	-3.560	0.0044				
LOC_Os07g44780					59.163	150.928	1.351	0.0193				
LOC_Os07g44890					72.814	455.418	2.645	0.0025	69.177	229.716	1.732	0.0067
LOC_Os07g44910					34.957	142.887	2.031	0.0025				
LOC_Os07g44920									39.778	216.185	2.442	0.0067

LOC_Os07g45260	3.737	18.113	2.277	0.0397	6.564	48.440	2.884	0.0025				
LOC_Os07g45570									7.646	25.908	1.761	0.0376
LOC_Os07g46480									24.883	3.408	-2.868	0.0324
LOC_Os07g46846					0.511	50.558	6.630	0.0025				
LOC_Os07g46860	0.000	2.088	14.350	0.0091								
LOC_Os07g46920					95.285	273.513	1.521	0.0061				
LOC_Os07g47790	0.853	99.002	6.859	0.0448								
LOC_Os07g47960									33.142	8.150	-2.024	0.0184
LOC_Os07g48030	50.652	190.705	1.913	0.0230								
LOC_Os07g48330	0.577	7.934	3.781	0.0091								
LOC_Os07g48410					42.374	14.536	-1.544	0.0025				
LOC_Os07g48450					35.099	9.461	-1.891	0.0025				
LOC_Os07g49000					7.208	0.083	-6.446	0.0025				
LOC_Os07g49140									2.534	0.107	-4.559	0.0067
LOC_Os08g01480					8.863	27.410	1.629	0.0146				
LOC_Os08g01520									0.000	0.343	11.746	0.0067
LOC_Os08g01940					6.381	26.850	2.073	0.0025				
LOC_Os08g02520					6.376	0.608	-3.391	0.0333				
LOC_Os08g03420	2.141	41.658	4.282	0.0091								
LOC_Os08g04180					22.951	98.472	2.101	0.0025				
LOC_Os08g04210									0.326	71.564	7.778	0.0067
LOC_Os08g04240									0.297	26.071	6.454	0.0067
LOC_Os08g04470					6.337	20.365	1.684	0.0469				
LOC_Os08g04540	0.000	0.933	13.187	0.0091	0.191	6.260	5.032	0.0146				
LOC_Os08g04560					5.038	14.953	1.569	0.0282				
LOC_Os08g04630					6.576	75.948	3.530	0.0025				
LOC_Os08g05900					0.000	0.586	12.517	0.0182				

LOC_Os08g06110									23.186	5.887	-1.978	0.0114
LOC_Os08g06280					38.950	97.619	1.326	0.0273				
LOC_Os08g07690									45.615	10.210	-2.160	0.0067
LOC_Os08g08100	0.273	12.643	5.534	0.0230								
LOC_Os08g09010									1.694	12.445	2.877	0.0244
LOC_Os08g10010	8.809	0.433	-4.347	0.0091								
LOC_Os08g10080					43.477	6.965	-2.642	0.0025				
LOC_Os08g10310					1.627	0.217	-2.905	0.0170	0.528	5.025	3.252	0.0114
LOC_Os08g12830					8.734	21.522	1.301	0.0227				
LOC_Os08g13440									2.386	18.857	2.983	0.0214
LOC_Os08g14570					30.080	109.802	1.868	0.0025				
LOC_Os08g15050					12.566	44.436	1.822	0.0133				
LOC_Os08g15650					15.186	60.916	2.004	0.0025				
LOC_Os08g19114	9.647	26.795	1.474	0.0345	8.629	49.723	2.527	0.0025				
LOC_Os08g19670					40.662	9.621	-2.079	0.0092				
LOC_Os08g23780									14.477	4.339	-1.738	0.0214
LOC_Os08g25780									1.069	0.000	-13.384	0.0114
LOC_Os08g28430					1.655	0.000	-14.015	0.0025				
LOC_Os08g28670									50.145	11.991	-2.064	0.0184
LOC_Os08g28880					1.642	10.201	2.635	0.0182				
LOC_Os08g29570					4.077	17.618	2.112	0.0025				
LOC_Os08g30020					34.612	91.638	1.405	0.0044				
LOC_Os08g30150					2.259	11.960	2.404	0.0092	1.276	11.829	3.213	0.0067
LOC_Os08g30210					1.261	16.884	3.743	0.0025	0.308	14.712	5.578	0.0301
LOC_Os08g30770					0.589	3.300	2.485	0.0061				
LOC_Os08g30910	0.174	1.114	2.680	0.0165								
LOC_Os08g31090					14.856	44.341	1.578	0.0159				

LOC_Os08g31980					6.720	2.044	-1.717	0.0273				
LOC_Os08g32500					5.278	1.068	-2.305	0.0448				
LOC_Os08g32960	0.626	6.178	3.303	0.0091					0.976	28.567	4.872	0.0444
LOC_Os08g33280									0.000	0.397	11.954	0.0067
LOC_Os08g33660									4.225	0.473	-3.158	0.0244
LOC_Os08g33720					23.954	55.622	1.215	0.0227				
LOC_Os08g33740					8.300	1.133	-2.873	0.0092				
LOC_Os08g33800					0.295	0.000	-11.529	0.0025				
LOC_Os08g34150	20.624	81.970	1.991	0.0165	17.483	187.086	3.420	0.0025	41.271	330.949	3.003	0.0067
LOC_Os08g34210	8.502	49.286	2.535	0.0091								
LOC_Os08g34790					8.606	26.949	1.647	0.0025				
LOC_Os08g35090					3.960	0.950	-2.059	0.0462				
LOC_Os08g35110					5.555	18.630	1.746	0.0261	11.017	67.449	2.614	0.0067
LOC_Os08g35190					63.817	13.771	-2.212	0.0025				
LOC_Os08g35740					64.793	143.781	1.150	0.0305				
LOC_Os08g35760	5.634	0.000	-15.782	0.0091								
LOC_Os08g36030	0.837	5.438	2.699	0.0448								
LOC_Os08g36040									48.597	11.773	-2.045	0.0352
LOC_Os08g36220					11.943	2.621	-2.188	0.0105				
LOC_Os08g36250					17.164	2.740	-2.647	0.0044				
LOC_Os08g36480	3.027	12.792	2.079	0.0345								
LOC_Os08g36590					10.397	3.287	-1.661	0.0411				
LOC_Os08g36910									0.240	3.855	4.005	0.0214
LOC_Os08g36920					0.943	10.832	3.522	0.0193	0.000	0.300	11.552	0.0067
LOC_Os08g37580									35.546	13.798	-1.365	0.0485
LOC_Os08g37660					31.861	84.078	1.400	0.0261				
LOC_Os08g38110					0.725	4.886	2.752	0.0293				

LOC_Os08g38210					17.632	53.729	1.608	0.0025				
LOC_Os08g38220					37.033	130.134	1.813	0.0025				
LOC_Os08g38270					48.218	155.024	1.685	0.0025				
LOC_Os08g39140					115.613	268.223	1.214	0.0261				
LOC_Os08g39330					439.912	58.297	-2.916	0.0025	332.345	43.864	-2.922	0.0067
LOC_Os08g39590	4.228	0.158	-4.745	0.0091	3.295	0.240	-3.779	0.0455				
LOC_Os08g39730					1.506	13.271	3.139	0.0061				
LOC_Os08g39810									0.390	2.990	2.939	0.0324
LOC_Os08g39850					0.051	1.684	5.048	0.0133				
LOC_Os08g40270					1.264	5.807	2.199	0.0133				
LOC_Os08g40380									41.242	9.257	-2.156	0.0423
LOC_Os08g40680	0.726	20.001	4.783	0.0091								
LOC_Os08g40690	0.776	302.800	8.609	0.0091					6.796	154.720	4.509	0.0067
LOC_Os08g40720	56.129	7.428	-2.918	0.0165	26.739	2.879	-3.215	0.0025				
LOC_Os08g40850					4.853	13.588	1.486	0.0411				
LOC_Os08g41290									283.375	92.490	-1.615	0.0214
LOC_Os08g41320					12.054	3.241	-1.895	0.0333				
LOC_Os08g41750					1.206	6.688	2.471	0.0344				
LOC_Os08g42470					5.575	0.059	-6.569	0.0363				
LOC_Os08g42530					20.267	6.865	-1.562	0.0044				
LOC_Os08g42960					11.613	54.262	2.224	0.0025				
LOC_Os08g43200					0.000	0.496	12.276	0.0025				
LOC_Os08g43210	0.388	42.877	6.788	0.0091								
LOC_Os08g43230									0.166	3.045	4.197	0.0485
LOC_Os08g43730					6.540	1.158	-2.497	0.0025				
LOC_Os08g44000					0.366	3.046	3.056	0.0078	0.470	3.654	2.958	0.0067
LOC_Os08g44840					5.319	0.702	-2.921	0.0105				

LOC_Os08g45170					2.108	0.151	-3.807	0.0383				
LOC_Os09g02250					9.240	2.598	-1.831	0.0250				
LOC_Os09g06464									0.000	0.696	12.764	0.0067
LOC_Os09g06770	1.008	16.816	4.060	0.0091								
LOC_Os09g08130					0.635	9.467	3.898	0.0025				
LOC_Os09g08190									0.488	3.993	3.032	0.0271
LOC_Os09g08390					48.260	96.982	1.007	0.0326				
LOC_Os09g10340	5.365	0.000	-15.711	0.0091	2.174	0.000	-14.408	0.0025				
LOC_Os09g11480									2.032	34.762	4.096	0.0067
LOC_Os09g13650									58.936	18.423	-1.678	0.0184
LOC_Os09g13930					3.727	0.386	-3.273	0.0182				
LOC_Os09g15420					4.800	18.740	1.965	0.0025				
LOC_Os09g16510	5.056	53.787	3.411	0.0091	8.190	85.246	3.380	0.0025	7.798	62.304	2.998	0.0067
LOC_Os09g16920	19.464	3.529	-2.464	0.0494	14.732	2.930	-2.330	0.0273				
LOC_Os09g17740					0.000	2.547	14.637	0.0025				
LOC_Os09g19650	2.432	0.000	-14.570	0.0091								
LOC_Os09g19790					68.986	361.792	2.391	0.0025				
LOC_Os09g19800									9.706	1.144	-3.085	0.0271
LOC_Os09g19930	2.291	0.081	-4.822	0.0091								
LOC_Os09g19970									0.991	7.241	2.870	0.0324
LOC_Os09g20700					0.637	0.000	-12.637	0.0025				
LOC_Os09g22510									1.475	13.433	3.187	0.0152
LOC_Os09g23560					0.627	4.526	2.852	0.0261				
LOC_Os09g23660					31.337	120.008	1.937	0.0025				
LOC_Os09g24580					12.882	81.585	2.663	0.0044				
LOC_Os09g25460					42.069	204.904	2.284	0.0025				
LOC_Os09g25570					7.126	1.172	-2.604	0.0025				

LOC_Os09g26210					0.133	3.455	4.697	0.0420				
LOC_Os09g26490					3.015	25.724	3.093	0.0092				
LOC_Os09g26780					10.571	151.231	3.839	0.0025				
LOC_Os09g27010					5.983	20.043	1.744	0.0182				
LOC_Os09g27230					33.760	12.670	-1.414	0.0440				
LOC_Os09g27650					5.685	1.350	-2.074	0.0363				
LOC_Os09g27820					91.432	27.207	-1.749	0.0025	66.285	14.128	-2.230	0.0067
LOC_Os09g28160					2.484	14.489	2.544	0.0025				
LOC_Os09g28340					10.364	1.019	-3.346	0.0025	10.441	1.318	-2.986	0.0114
LOC_Os09g28550					25.318	5.453	-2.215	0.0025				
LOC_Os09g28580	3.275	41.637	3.668	0.0091								
LOC_Os09g28690									179.141	65.401	-1.454	0.0152
LOC_Os09g28810					5.902	0.932	-2.663	0.0044				
LOC_Os09g28880					1.799	0.102	-4.139	0.0025				
LOC_Os09g28910					9.389	48.473	2.368	0.0025				
LOC_Os09g29239									50.403	167.359	1.731	0.0324
LOC_Os09g29300					1.036	21.117	4.349	0.0025	0.943	10.413	3.465	0.0114
LOC_Os09g29310					16.591	70.445	2.086	0.0025				
LOC_Os09g29660					0.860	5.995	2.801	0.0261				
LOC_Os09g29670					0.205	1.824	3.152	0.0025				
LOC_Os09g29710					0.913	298.921	8.356	0.0025				
LOC_Os09g30490	4.862	92.064	4.243	0.0091	4.279	21.421	2.324	0.0025	5.901	63.320	3.424	0.0114
LOC_Os09g31000									0.361	14.970	5.373	0.0114
LOC_Os09g31031	54.390	243.009	2.160	0.0091								
LOC_Os09g31040	1.129	124.038	6.780	0.0091					4.365	40.329	3.208	0.0324
LOC_Os09g32320									11.893	60.174	2.339	0.0067
LOC_Os09g32570					0.289	2.715	3.233	0.0146				

LOC_Os09g32670					50.693	17.626	-1.524	0.0193				
LOC_Os09g33555					7.083	1.209	-2.550	0.0383				
LOC_Os09g33580					34.510	2.038	-4.082	0.0025				
LOC_Os09g33640					0.853	9.835	3.527	0.0182				
LOC_Os09g33650					1.306	15.208	3.541	0.0025				
LOC_Os09g33680									4.330	20.453	2.240	0.0067
LOC_Os09g34150					0.569	5.048	3.150	0.0025				
LOC_Os09g34960	11.860	1.252	-3.244	0.0345	12.274	1.311	-3.227	0.0025				
LOC_Os09g35940					7.920	0.214	-5.211	0.0216				
LOC_Os09g36560					0.978	0.000	-13.256	0.0025				
LOC_Os09g36600	12.805	50.395	1.977	0.0345								
LOC_Os09g36700	7.988	0.263	-4.924	0.0296					1.353	13.368	3.304	0.0324
LOC_Os09g37080					1.773	10.522	2.569	0.0404				
LOC_Os09g37410					12.407	1.154	-3.426	0.0092				
LOC_Os09g37420					7.982	1.328	-2.587	0.0282				
LOC_Os09g37600	5.788	0.760	-2.930	0.0296	6.008	0.916	-2.713	0.0333	26.040	3.264	-2.996	0.0067
LOC_Os09g37910									42.875	7.415	-2.532	0.0067
LOC_Os09g38130					11.660	41.349	1.826	0.0025				
LOC_Os09g38850					0.697	3.207	2.201	0.0133				
LOC_Os09g38920					27.466	69.266	1.335	0.0182				
LOC_Os09g39430					25.406	5.240	-2.278	0.0025				
LOC_Os09g39770					39.843	109.690	1.461	0.0227				
LOC_Os10g01930									22.021	4.220	-2.384	0.0067
LOC_Os10g02880					0.353	10.656	4.918	0.0105				
LOC_Os10g04090					6.353	1.649	-1.946	0.0092				
LOC_Os10g05600					10.483	1.367	-2.939	0.0170				
LOC_Os10g05660					7.578	49.855	2.718	0.0025				

LOC_Os10g05950									63.087	3.263	-4.273	0.0067
LOC_Os10g05970									27.215	1.022	-4.734	0.0067
LOC_Os10g05980									48.261	0.884	-5.771	0.0067
LOC_Os10g05990									46.364	1.870	-4.632	0.0114
LOC_Os10g06000									19.945	0.916	-4.445	0.0067
LOC_Os10g07229					30.022	93.316	1.636	0.0025				
LOC_Os10g07290					24.934	2.559	-3.285	0.0025				
LOC_Os10g08580					2.051	25.547	3.639	0.0025	2.571	21.700	3.077	0.0067
LOC_Os10g09110					1.454	12.655	3.122	0.0105				
LOC_Os10g11500					235.689	1970.656	3.064	0.0025				
LOC_Os10g11980					2.029	0.000	-14.309	0.0025				
LOC_Os10g18370					0.394	2.760	2.808	0.0238				
LOC_Os10g20610	0.000	0.720	12.813	0.0091								
LOC_Os10g21670									241.656	67.667	-1.836	0.0271
LOC_Os10g23050					60.517	8.488	-2.834	0.0025				
LOC_Os10g23090					47.132	16.154	-1.545	0.0078				
LOC_Os10g25180					4.263	11.555	1.439	0.0455				
LOC_Os10g25230	0.522	53.105	6.667	0.0091	1.988	70.034	5.139	0.0025	0.510	13.072	4.680	0.0485
LOC_Os10g25290					13.199	145.738	3.465	0.0025				
LOC_Os10g25340					9.823	63.572	2.694	0.0025				
LOC_Os10g25400					0.498	15.493	4.960	0.0025				
LOC_Os10g26500					22.942	4.934	-2.217	0.0061				
LOC_Os10g26700	1.101	152.977	7.119	0.0091								
LOC_Os10g28080					29.794	120.877	2.021	0.0455				
LOC_Os10g28200					16.669	44.796	1.426	0.0025	14.770	57.779	1.968	0.0067
LOC_Os10g28210					74.901	27.134	-1.465	0.0227				
LOC_Os10g28350									7.723	33.291	2.108	0.0271

LOC_Os10g30390					0.000	5.283	15.689	0.0025				
LOC_Os10g30450					45.577	99.602	1.128	0.0495				
LOC_Os10g31420									1.198	22.024	4.200	0.0067
LOC_Os10g33040					1.081	10.249	3.244	0.0025				
LOC_Os10g33060									6.351	0.806	-2.979	0.0152
LOC_Os10g33370					1.528	0.117	-3.704	0.0044				
LOC_Os10g33810					1.020	8.285	3.022	0.0170				
LOC_Os10g33900	8.920	0.992	-3.169	0.0345								
LOC_Os10g34700					3.995	0.292	-3.774	0.0025				
LOC_Os10g37190					44.263	178.396	2.011	0.0025				
LOC_Os10g38090									15.805	3.690	-2.099	0.0152
LOC_Os10g38730					15.747	102.118	2.697	0.0025				
LOC_Os10g38880					42.346	114.980	1.441	0.0216	39.179	134.594	1.780	0.0464
LOC_Os10g39010					6.759	21.956	1.700	0.0170				
LOC_Os10g39140					18.224	61.383	1.752	0.0025				
LOC_Os10g39210					0.816	0.000	-12.995	0.0025				
LOC_Os10g39840									15.745	1.166	-3.755	0.0067
LOC_Os10g40360					19.238	118.286	2.620	0.0025				
LOC_Os10g40430					728.697	65.518	-3.475	0.0025	224.041	34.403	-2.703	0.0067
LOC_Os10g40530					1487.755	366.371	-2.022	0.0044				
LOC_Os10g40550					2.161	7.039	1.704	0.0462				
LOC_Os10g40600					40.201	16.795	-1.259	0.0411				
LOC_Os10g40614					35.270	2.894	-3.608	0.0025				
LOC_Os10g40810	14.437	0.990	-3.867	0.0091	11.493	0.128	-6.487	0.0025				
LOC_Os10g41130	11.127	46.382	2.060	0.0397	14.408	110.127	2.934	0.0025	13.540	67.092	2.309	0.0184
LOC_Os10g41330					7.486	28.140	1.910	0.0193				
LOC_Os10g41930					5.406	16.099	1.574	0.0326				

LOC_Os10g42110					11.075	2.936	-1.915	0.0159				
LOC_Os10g42190					44.363	15.073	-1.557	0.0120				
LOC_Os10g42430					54.089	151.178	1.483	0.0044				
LOC_Os10g42610					48.901	15.147	-1.691	0.0044				
LOC_Os10g43060					17.969	37.717	1.070	0.0462				
LOC_Os11g01040					2.656	26.261	3.306	0.0025				
LOC_Os11g02080					218.492	97.983	-1.157	0.0448				
LOC_Os11g02350					111.177	31.620	-1.814	0.0025				
LOC_Os11g02379									10.074	2.198	-2.196	0.0301
LOC_Os11g02480					1.723	10.778	2.645	0.0025				
LOC_Os11g02610					1.267	19.825	3.968	0.0025	1.356	42.620	4.974	0.0067
LOC_Os11g03240					12.048	55.060	2.192	0.0025	11.582	39.100	1.755	0.0376
LOC_Os11g03440					1.159	23.436	4.338	0.0025				
LOC_Os11g03484					1.571	18.722	3.575	0.0025	1.359	10.150	2.901	0.0152
LOC_Os11g03970					21.116	47.119	1.158	0.0227				
LOC_Os11g05010					191.504	66.472	-1.527	0.0061				
LOC_Os11g05290					66.475	4.701	-3.822	0.0025				
LOC_Os11g05380					0.941	9.856	3.388	0.0025	0.693	6.480	3.226	0.0067
LOC_Os11g05470									16.144	57.117	1.823	0.0324
LOC_Os11g05770					9.663	3.001	-1.687	0.0282				
LOC_Os11g07460					6.247	0.722	-3.114	0.0078				
LOC_Os11g07912					9.326	2.340	-1.995	0.0227				
LOC_Os11g07960					87.503	24.394	-1.843	0.0025				
LOC_Os11g08140									0.750	0.000	-12.873	0.0067
LOC_Os11g09010					11.025	41.939	1.928	0.0025				
LOC_Os11g10460					22.220	4.483	-2.309	0.0044				
LOC_Os11g10590					504.907	95.740	-2.399	0.0025				

LOC_Os11g11000					7.266	18.570	1.354	0.0170				
LOC_Os11g11650					1.099	0.093	-3.560	0.0469				
LOC_Os11g11960									1.852	10.984	2.568	0.0067
LOC_Os11g13710					3.982	29.530	2.891	0.0025	5.257	48.083	3.193	0.0376
LOC_Os11g14070					8.895	1.363	-2.706	0.0353				
LOC_Os11g14140					414.057	142.330	-1.541	0.0061				
LOC_Os11g15230					13.245	37.434	1.499	0.0182	18.250	116.812	2.678	0.0067
LOC_Os11g15340					3.201	12.515	1.967	0.0105	4.656	45.945	3.303	0.0067
LOC_Os11g15570					3.564	23.714	2.734	0.0025				
LOC_Os11g16580					3.907	22.018	2.495	0.0025	4.248	21.765	2.357	0.0067
LOC_Os11g17720									1.736	7.487	2.109	0.0423
LOC_Os11g18730									392.304	82.346	-2.252	0.0067
LOC_Os11g18870									0.000	0.305	11.577	0.0244
LOC_Os11g20040									24.373	3.638	-2.744	0.0067
LOC_Os11g20090									629.567	186.641	-1.754	0.0114
LOC_Os11g20160									665.658	168.816	-1.979	0.0464
LOC_Os11g24060	0.558	5.482	3.296	0.0397	1.133	25.013	4.465	0.0025	1.076	16.746	3.960	0.0067
LOC_Os11g24070	98.491	4.234	-4.540	0.0091	30.040	3.232	-3.216	0.0025				
LOC_Os11g25990	0.884	10.769	3.607	0.0091	1.095	11.400	3.380	0.0061	2.507	64.519	4.685	0.0067
LOC_Os11g26340									0.298	0.000	-11.539	0.0271
LOC_Os11g26790	13.322	0.971	-3.778	0.0091								
LOC_Os11g26880					6.518	1.303	-2.322	0.0250				
LOC_Os11g26990					2.477	0.597	-2.053	0.0469				
LOC_Os11g28530					0.712	4.744	2.736	0.0105				
LOC_Os11g29290	3.542	39.926	3.495	0.0091	6.133	134.011	4.450	0.0025	1.625	14.827	3.190	0.0244
LOC_Os11g30360					80.517	263.723	1.712	0.0025	61.645	206.975	1.747	0.0214
LOC_Os11g33270					494.465	45.327	-3.447	0.0025	224.615	31.861	-2.818	0.0067

LOC_Os11g34020					9.000	30.199	1.746	0.0485				
LOC_Os11g35080					5.838	25.942	2.152	0.0105				
LOC_Os11g35500									0.274	1.958	2.835	0.0184
LOC_Os11g36090									0.135	1.032	2.936	0.0376
LOC_Os11g37230					14.929	36.939	1.307	0.0404				
LOC_Os11g37670					10.120	0.259	-5.289	0.0105				
LOC_Os11g37700									13.908	3.349	-2.054	0.0067
LOC_Os11g37940					39.966	136.484	1.772	0.0170				
LOC_Os11g37950					33.858	327.447	3.274	0.0025				
LOC_Os11g37970					15.308	254.269	4.054	0.0025				
LOC_Os11g38810					4.214	26.958	2.678	0.0025				
LOC_Os11g39020									4.071	14.241	1.807	0.0352
LOC_Os11g40590					159.122	47.156	-1.755	0.0120				
LOC_Os11g41680					50.588	20.099	-1.332	0.0273	51.175	13.099	-1.966	0.0067
LOC_Os11g42200	0.000	1.428	13.802	0.0091								
LOC_Os11g42500									193.889	36.372	-2.414	0.0067
LOC_Os11g42550									46.547	5.664	-3.039	0.0067
LOC_Os11g43750					43.952	12.894	-1.769	0.0061				
LOC_Os11g43860					10.470	58.786	2.489	0.0025				
LOC_Os11g44810					1349.208	566.147	-1.253	0.0273				
LOC_Os11g44950					11.497	3.235	-1.829	0.0305				
LOC_Os11g45280									0.574	14.124	4.622	0.0067
LOC_Os11g45400					0.651	13.052	4.325	0.0025				
LOC_Os11g45740					7.938	35.838	2.175	0.0025				
LOC_Os11g46810					0.000	0.323	11.659	0.0025				
LOC_Os11g46860					0.325	34.055	6.712	0.0025	0.963	144.997	7.234	0.0067
LOC_Os11g46870					0.000	0.185	10.856	0.0025				

LOC_Os11g46880									0.075	3.093	5.362	0.0244
LOC_Os11g47500	10.633	114.186	3.425	0.0091								
LOC_Os11g47510	0.430	28.606	6.055	0.0091					3.533	66.819	4.241	0.0114
LOC_Os11g47520	1.826	16.832	3.204	0.0091					2.967	63.337	4.416	0.0067
LOC_Os11g47530									0.900	20.191	4.488	0.0402
LOC_Os11g47550									0.386	60.932	7.302	0.0114
LOC_Os11g47560									0.790	35.656	5.496	0.0067
LOC_Os11g47570	0.811	12.794	3.980	0.0091					2.581	23.752	3.202	0.0184
LOC_Os11g47580									0.938	24.393	4.701	0.0067
LOC_Os11g47600									4.332	53.729	3.633	0.0214
LOC_Os12g01030					3.427	30.568	3.157	0.0025				
LOC_Os12g01380					18.239	40.033	1.134	0.0485				
LOC_Os12g02300					125.832	32.079	-1.972	0.0025	109.795	19.129	-2.521	0.0184
LOC_Os12g02420					3.096	15.988	2.369	0.0025				
LOC_Os12g02530					0.531	6.539	3.621	0.0227	0.403	14.755	5.193	0.0244
LOC_Os12g03150	1.072	12.195	3.508	0.0091	0.884	14.034	3.988	0.0025				
LOC_Os12g03200					20.255	46.934	1.212	0.0315				
LOC_Os12g03230					3.669	19.491	2.410	0.0326				
LOC_Os12g03240					1.158	13.151	3.505	0.0044				
LOC_Os12g04500									20.819	5.330	-1.966	0.0444
LOC_Os12g06030	0.000	1.637	13.999	0.0091								
LOC_Os12g06220					39.437	106.311	1.431	0.0238				
LOC_Os12g06340									138.602	47.888	-1.533	0.0114
LOC_Os12g07030					1.191	78.989	6.051	0.0025				
LOC_Os12g07160					13.445	4.860	-1.468	0.0411				
LOC_Os12g07810					0.266	1.948	2.873	0.0170				
LOC_Os12g08760									81.862	11.963	-2.775	0.0067

LOC_Os12g08810					34.776	220.904	2.667	0.0025	25.713	141.361	2.459	0.0067
LOC_Os12g08920					4.788	0.591	-3.018	0.0092				
LOC_Os12g10670					2.094	0.144	-3.864	0.0025				
LOC_Os12g11770									0.000	1.150	13.489	0.0244
LOC_Os12g13640					6.409	19.857	1.632	0.0462				
LOC_Os12g14070					21.012	52.641	1.325	0.0078				
LOC_Os12g14220					8.134	1.038	-2.970	0.0411				
LOC_Os12g14580					0.553	0.000	-12.433	0.0025				
LOC_Os12g16310					1.460	0.228	-2.675	0.0420				
LOC_Os12g17430					4.164	13.609	1.709	0.0315				
LOC_Os12g18640					0.918	6.027	2.715	0.0025				
LOC_Os12g19180					2.241	0.207	-3.435	0.0025				
LOC_Os12g20390					18.435	55.264	1.584	0.0333				
LOC_Os12g24020					338.943	80.684	-2.071	0.0025				
LOC_Os12g24320					14.662	2.386	-2.620	0.0044				
LOC_Os12g24390					20.539	3.860	-2.412	0.0044				
LOC_Os12g27350					288.131	67.731	-2.089	0.0025				
LOC_Os12g28590					11.984	65.735	2.456	0.0025				
LOC_Os12g28710					4.468	23.242	2.379	0.0025				
LOC_Os12g29400					14.770	50.254	1.767	0.0182				
LOC_Os12g30790					0.000	0.439	12.099	0.0025				
LOC_Os12g31520	0.910	28.822	4.984	0.0091								
LOC_Os12g32760									4.443	34.436	2.954	0.0067
LOC_Os12g35610	3.231	66.765	4.369	0.0091								
LOC_Os12g36110					6.259	18.098	1.532	0.0205				
LOC_Os12g36630					8.237	1.385	-2.572	0.0469				
LOC_Os12g36660					0.627	0.000	-12.614	0.0025				

LOC_Os12g36810					19.225	2.629	-2.870	0.0025				
LOC_Os12g36830	6.370	62.840	3.302	0.0091								
LOC_Os12g36850	2.392	31.072	3.700	0.0230	40.813	158.016	1.953	0.0282				
LOC_Os12g37690					0.505	12.057	4.579	0.0025				
LOC_Os12g38140	85.453	0.850	-6.651	0.0091	69.594	3.177	-4.453	0.0044				
LOC_Os12g38400					14.024	3.011	-2.220	0.0025				
LOC_Os12g38750									4.740	15.473	1.707	0.0324
LOC_Os12g38770	0.494	11.335	4.521	0.0091								
LOC_Os12g39380					13.658	3.356	-2.025	0.0025				
LOC_Os12g40180	6.482	311.502	5.587	0.0091								
LOC_Os12g40330	5.719	26.448	2.209	0.0296								
LOC_Os12g41590									0.737	9.242	3.648	0.0067
LOC_Os12g41720					18.396	56.437	1.617	0.0105				
LOC_Os12g42250					5.548	21.848	1.978	0.0105	6.008	23.849	1.989	0.0184
LOC_Os12g42300					0.746	4.348	2.543	0.0025	0.741	7.858	3.406	0.0067
LOC_Os12g42910					2.742	12.189	2.152	0.0282				
LOC_Os12g43130					0.449	2.284	2.347	0.0261	0.560	12.396	4.469	0.0067
LOC_Os12g43363					11.995	2.324	-2.368	0.0092				
LOC_Os12g43380					50.619	296.932	2.552	0.0205				
LOC_Os12g43490					57.336	168.968	1.559	0.0170				
LOC_Os12g43640					20.249	67.308	1.733	0.0025				
LOC_Os12g44050									0.000	0.397	11.954	0.0067
LOC_Os12g44170					9.768	34.436	1.818	0.0025				
LOC_Os12g44180					9.730	23.267	1.258	0.0353				
LOC_Os12g44270	12.590	0.000	-16.942	0.0091	5.462	0.000	-15.737	0.0025				
LOC_Os12g44370					2.614	12.263	2.230	0.0025				

Table S3. Gene ontology (GO) enrichment analysis of DEGs after light treatment.

Light treatment time	GO ID	Description	Ratio_in_study	Ratio_in_pop	p_uncorrected	p_bonferroni
20 min	GO:0009889	regulation of biosynthetic process	29/192	1999/55958	6.38E-11	6.73E-08
	GO:0006355	regulation of transcription	41/192	2458/55958	9.13E-11	9.62E-08
	GO:2001141	regulation of RNA biosynthetic process	26/192	1685/55958	1.87E-10	1.97E-07
	GO:0031326	regulation of cellular biosynthetic process	28/192	1983/55958	2.60E-10	2.74E-07
	GO:0010468	regulation of gene expression	27/192	2062/55958	2.81E-09	2.97E-06
	GO:0010556	regulation of macromolecule biosynthetic process	26/192	1947/55958	3.82E-09	4.02E-06
	GO:2000112	regulation of cellular macromolecule biosynthetic proce	26/192	1947/55958	3.82E-09	4.02E-06
	GO:0055114	oxidation-reduction process	27/192	2173/55958	8.45E-09	8.91E-06
	GO:0044255	cellular lipid metabolic process	14/192	773/55958	5.59E-07	0.000589
	GO:0006629	lipid metabolic process	16/192	1030/55958	6.51E-07	0.000686
	GO:0044281	small molecule metabolic process	34/192	3968/55958	7.00E-07	0.000738
	GO:0032787	monocarboxylic acid metabolic process	10/192	405/55958	1.62E-06	0.00171
	GO:0006952	defense response	16/192	1149/55958	2.66E-06	0.0028
	GO:0034605	cellular response to heat	3/192	9/55958	3.29E-06	0.00347
	GO:0043436	oxoacid metabolic process	26/192	2774/55958	3.50E-06	0.00368
	GO:0006082	organic acid metabolic process	26/192	2776/55958	3.54E-06	0.00373
	GO:0044711	single-organism biosynthetic process	19/192	1617/55958	3.58E-06	0.00377
	GO:0044707	single-multicellular organism process	13/192	812/55958	5.45E-06	0.00575
	GO:0032501	multicellular organismal process	13/192	818/55958	5.90E-06	0.00622
	GO:0034059	response to anoxia	3/192	12/55958	8.55E-06	0.00901
	GO:0019752	carboxylic acid metabolic process	25/192	2741/55958	8.74E-06	0.00922

	GO:0044283	small molecule biosynthetic process	12/192	726/55958	9.19E-06	0.00969
	GO:0009628	response to abiotic stimulus	17/192	1435/55958	1.07E-05	0.0113
	GO:0044767	single-organism developmental process	17/192	1456/55958	1.29E-05	0.0136
	GO:0006631	fatty acid metabolic process	7/192	234/55958	1.81E-05	0.0191
	GO:0007275	multicellular organismal development	7/192	241/55958	2.19E-05	0.0231
	GO:0046394	carboxylic acid biosynthetic process	10/192	574/55958	3.34E-05	0.0352
	GO:0016053	organic acid biosynthetic process	10/192	574/55958	3.34E-05	0.0352
	GO:0006040	amino sugar metabolic process	5/192	107/55958	3.61E-05	0.0381
	GO:0008610	lipid biosynthetic process	9/192	465/55958	3.70E-05	0.039
	GO:0032502	developmental process	17/192	1611/55958	4.59E-05	0.0483
	GO:0009416	response to light stimulus	35/661	441/55958	3.23E-11	5.40E-08
	GO:0009725	response to hormone	40/661	834/55958	4.42E-11	7.38E-08
	GO:0009719	response to endogenous stimulus	41/661	878/55958	4.66E-11	7.78E-08
	GO:0009628	response to abiotic stimulus	70/661	1435/55958	5.70E-11	9.53E-08
	GO:2001141	regulation of RNA biosynthetic process	55/661	1685/55958	5.99E-11	1.00E-07
	GO:0009314	response to radiation	35/661	457/55958	7.03E-11	1.17E-07
	GO:0044283	small molecule biosynthetic process	33/661	726/55958	7.67E-11	1.28E-07
	GO:0006629	lipid metabolic process	55/661	1030/55958	8.69E-11	1.45E-07
	GO:0044255	cellular lipid metabolic process	42/661	773/55958	9.41E-11	1.57E-07
	GO:0010033	response to organic substance	48/661	1033/55958	9.47E-11	1.58E-07
	GO:0010468	regulation of gene expression	62/661	2062/55958	9.47E-11	1.58E-07
	GO:0046394	carboxylic acid biosynthetic process	30/661	574/55958	9.50E-11	1.59E-07
	GO:0016053	organic acid biosynthetic process	30/661	574/55958	9.50E-11	1.59E-07
	GO:0043436	oxoacid metabolic process	77/661	2774/55958	9.55E-11	1.60E-07

60 min	GO:0009889	regulation of biosynthetic process	65/661	1999/55958	9.72E-11	1.62E-07
	GO:0006082	organic acid metabolic process	77/661	2776/55958	9.78E-11	1.63E-07
	GO:0008610	lipid biosynthetic process	30/661	465/55958	1.02E-10	1.71E-07
	GO:0042221	response to chemical	62/661	1711/55958	1.04E-10	1.75E-07
	GO:1901700	response to oxygen-containing compound	47/661	949/55958	1.10E-10	1.83E-07
	GO:0072330	monocarboxylic acid biosynthetic process	20/661	241/55958	1.16E-10	1.94E-07
	GO:0044711	single-organism biosynthetic process	56/661	1617/55958	1.22E-10	2.04E-07
	GO:0019752	carboxylic acid metabolic process	77/661	2741/55958	1.40E-10	2.33E-07
	GO:0006355	regulation of transcription	95/661	2458/55958	1.63E-10	2.72E-07
	GO:0031326	regulation of cellular biosynthetic process	65/661	1983/55958	1.73E-10	2.88E-07
	GO:0055114	oxidation-reduction process	91/661	2173/55958	1.89E-10	3.16E-07
	GO:2000112	regulation of cellular macromolecule biosynthetic proce	59/661	1947/55958	2.45E-10	4.10E-07
	GO:0010556	regulation of macromolecule biosynthetic process	59/661	1947/55958	2.45E-10	4.10E-07
	GO:0080167	response to karrikin	15/661	147/55958	2.92E-10	4.89E-07
	GO:0006633	fatty acid biosynthetic process	16/661	184/55958	8.12E-10	1.36E-06
	GO:0032787	monocarboxylic acid metabolic process	23/661	405/55958	9.08E-10	1.52E-06
	GO:0043565	sequence-specific DNA binding	26/661	521/55958	1.15E-09	1.92E-06
	GO:0051707	response to other organism	27/661	564/55958	1.35E-09	2.26E-06
	GO:0051704	multi-organism process	28/661	615/55958	2.03E-09	3.39E-06
	GO:0031408	oxylipin biosynthetic process	8/661	32/55958	2.98E-09	4.98E-06
	GO:0031407	oxylipin metabolic process	8/661	32/55958	2.98E-09	4.98E-06
	GO:0006631	fatty acid metabolic process	17/661	234/55958	3.80E-09	6.35E-06
	GO:0009607	response to biotic stimulus	27/661	603/55958	5.59E-09	9.34E-06
	GO:0006970	response to osmotic stress	24/661	491/55958	7.45E-09	1.24E-05

GO:0009651	response to salt stress	23/661	455/55958	8.20E-09	1.37E-05
GO:0016101	diterpenoid metabolic process	8/661	43/55958	3.66E-08	6.12E-05
GO:1901564	organonitrogen compound metabolic process	80/661	3610/55958	6.79E-08	0.000113
GO:0033993	response to lipid	21/661	435/55958	7.90E-08	0.000132
GO:0009642	response to light intensity	10/661	89/55958	1.09E-07	0.000182
GO:0009617	response to bacterium	16/661	266/55958	1.50E-07	0.00025
GO:0009411	response to UV	10/661	93/55958	1.66E-07	0.000277
GO:0014070	response to organic cyclic compound	14/661	204/55958	1.81E-07	0.000302
GO:0009753	response to jasmonic acid	11/661	121/55958	2.27E-07	0.000379
GO:0010035	response to inorganic substance	29/661	817/55958	2.28E-07	0.000381
GO:0016102	diterpenoid biosynthetic process	6/661	24/55958	2.98E-07	0.000498
GO:0008299	isoprenoid biosynthetic process	11/661	127/55958	3.71E-07	0.00062
GO:0006720	isoprenoid metabolic process	12/661	159/55958	4.92E-07	0.000822
GO:0006721	terpenoid metabolic process	11/661	137/55958	7.94E-07	0.00133
GO:0044712	single-organism catabolic process	32/661	1025/55958	9.09E-07	0.00152
GO:0010224	response to UV-B	8/661	66/55958	1.14E-06	0.00191
GO:0009751	response to salicylic acid	9/661	96/55958	2.21E-06	0.00369
GO:0034605	cellular response to heat	4/661	9/55958	2.32E-06	0.00388
GO:0009611	response to wounding	12/661	187/55958	2.73E-06	0.00456
GO:0052314	phytoalexin metabolic process	5/661	20/55958	3.03E-06	0.00507
GO:0009404	toxin metabolic process	5/661	20/55958	3.03E-06	0.00507
GO:0009408	response to heat	12/661	189/55958	3.05E-06	0.00509
GO:0009644	response to high light intensity	7/661	54/55958	3.40E-06	0.00569
GO:1902221	erythrose 4-phosphate/phosphoenolpyruvate family ami	9/661	102/55958	3.66E-06	0.00612

	GO:0006558	L-phenylalanine metabolic process	9/661	102/55958	3.66E-06	0.00612
	GO:0098542	defense response to other organism	17/661	383/55958	4.14E-06	0.00692
	GO:0097305	response to alcohol	17/661	384/55958	4.29E-06	0.00716
	GO:0016114	terpenoid biosynthetic process	9/661	104/55958	4.30E-06	0.00719
	GO:0006952	defense response	33/661	1149/55958	4.68E-06	0.00782
	GO:0019748	secondary metabolic process	11/661	167/55958	5.50E-06	0.0092
	GO:0006570	tyrosine metabolic process	10/661	137/55958	5.91E-06	0.00988
	GO:0009415	response to water	13/661	239/55958	6.52E-06	0.0109
	GO:0005975	carbohydrate metabolic process	36/661	1353/55958	8.73E-06	0.0146
	GO:0009266	response to temperature stimulus	18/661	451/55958	9.35E-06	0.0156
	GO:0009056	catabolic process	39/661	1530/55958	1.00E-05	0.0168
	GO:0080022	primary root development	4/661	13/55958	1.27E-05	0.0212
	GO:0071492	cellular response to UV-A	3/661	5/55958	1.61E-05	0.0269
	GO:0070141	response to UV-A	3/661	5/55958	1.61E-05	0.0269
	GO:0009414	response to water deprivation	12/661	227/55958	1.95E-05	0.0325
	GO:0006855	drug transmembrane transport	7/661	70/55958	1.96E-05	0.0327
	GO:0006520	cellular amino acid metabolic process	52/661	2342/55958	2.05E-05	0.0342
	GO:0051501	diterpene phytoalexin metabolic process	4/661	15/55958	2.37E-05	0.0397
	GO:0071489	cellular response to red or far red light	4/661	15/55958	2.37E-05	0.0397
	GO:0006979	response to oxidative stress	18/661	484/55958	2.39E-05	0.0399
	GO:1901575	organic substance catabolic process	37/661	1481/55958	2.45E-05	0.0409
	GO:0009637	response to blue light	7/661	73/55958	2.58E-05	0.0431
	GO:0009628	response to abiotic stimulus	33/320	1435/55958	8.17E-11	1.11E-07
	GO:0005975	carbohydrate metabolic process	35/320	1353/55958	8.48E-11	1.16E-07

360 min	GO:0055114	oxidation-reduction process	49/320	2173/55958	1.00E-10	1.37E-07
	GO:0050896	response to stimulus	74/320	4632/55958	1.26E-10	1.72E-07
	GO:0009416	response to light stimulus	17/320	441/55958	1.03E-09	1.40E-06
	GO:0009314	response to radiation	17/320	457/55958	1.75E-09	2.39E-06
	GO:0080167	response to karrikin	10/320	147/55958	1.55E-08	2.11E-05
	GO:1901700	response to oxygen-containing compound	20/320	949/55958	7.70E-07	0.00105
	GO:0009739	response to gibberellin	6/320	64/55958	1.89E-06	0.00258
	GO:0010033	response to organic substance	20/320	1033/55958	2.77E-06	0.00378
	GO:0042221	response to chemical	26/320	1711/55958	7.50E-06	0.0102
	GO:0009725	response to hormone	17/320	834/55958	8.06E-06	0.011
	GO:0044042	glucan metabolic process	12/320	443/55958	1.14E-05	0.0156
	GO:0006073	cellular glucan metabolic process	12/320	443/55958	1.14E-05	0.0156
	GO:0051716	cellular response to stimulus	23/320	1440/55958	1.17E-05	0.0159
	GO:0009719	response to endogenous stimulus	17/320	878/55958	1.55E-05	0.0212
	GO:0044723	single-organism carbohydrate metabolic process	17/320	902/55958	2.18E-05	0.0298
	GO:0044264	cellular polysaccharide metabolic process	12/320	480/55958	2.51E-05	0.0343
	GO:0006979	response to oxidative stress	12/320	484/55958	2.72E-05	0.0371
	GO:0006629	lipid metabolic process	18/320	1030/55958	3.37E-05	0.0459

Table S4. KEGG enrichment analysis of DEGs at 60 minutes after light treatment.

Terms	Database	KEGG Id	Sample number	Background number	P-Value	Corrected P-Value
Diterpenoid biosynthesis	KEGG PATHWAY	ko00904	8	42	1.98E-06	0.000211507
Plant hormone signal transduction	KEGG PATHWAY	ko04075	17	260	6.29E-06	0.000336675
Phenylpropanoid biosynthesis	KEGG PATHWAY	ko00940	12	163	5.10E-05	0.00181939
α-Linolenic acid metabolism	KEGG PATHWAY	ko00592	6	46	0.000256	0.006856086
Linoleic acid metabolism	KEGG PATHWAY	ko00591	4	20	0.000693	0.014508197
Limonene and pinene degradation	KEGG PATHWAY	ko00903	4	21	0.000814	0.014508197
Phenylalanine metabolism	KEGG PATHWAY	ko00360	5	45	0.001631	0.024936536
Stilbenoid, diarylheptanoid and gingerol biosynthesis	KEGG PATHWAY	ko00945	4	28	0.002105	0.028149029

Table S5. Expression profiles of genes related to phytohormone signaling and biosynthesis.

Types	Names	Genes	20 minutes				60 minutes				360 minutes			
			Dark (FPKM)	Light (FPKM)	Log2(fold_change)	q_value	Dark (FPKM)	Light (FPKM)	Log2(fold_change)	q_value	Dark (FPKM)	Light (FPKM)	Log2(fold_change)	q_value
Phytohormone signaling	Auxin	LOC_Os01g12160					6.16	1.12	-2.46	0.0061				
	Auxin	LOC_Os01g57610					1.83	0.24	-2.92	0.0105				
	Auxin	LOC_Os02g24700					31.33	0.34	-6.51	0.0025				
	Auxin	LOC_Os06g04590									23.50	3.91	-2.59	0.0423
	Auxin	LOC_Os08g02520					6.38	0.61	-3.39	0.0333				
	Cytokinin	LOC_Os05g09410									8.38	52.39	2.65	0.0067
	GA	LOC_Os01g03890									4.08	32.67	3.00	0.0352
	GA	LOC_Os01g15340									5.74	29.29	2.35	0.0067
	GA	LOC_Os02g10860					1.61	18.45	3.52	0.0025	3.09	14.13	2.19	0.0444
	GA	LOC_Os01g51260									0.46	0.00	-12.18	0.0067
	GA	LOC_Os08g33660									4.22	0.47	-3.16	0.0244
	ABA	LOC_Os03g27280					3.28	10.74	1.71	0.0238	2.11	11.76	2.48	0.0244
	JA	LOC_Os05g50890					20.62	154.28	2.90	0.0025				
	JA	LOC_Os04g32480					0.80	7.80	3.28	0.0092				
	JA	LOC_Os07g42370					190.25	1124.54	2.56	0.0025	155.05	512.01	1.72	0.0152
	JA	LOC_Os09g26780					10.57	151.23	3.84	0.0025				
	JA	LOC_Os03g08310					1.13	12.46	3.46	0.0044				
	JA	LOC_Os03g08320					11.52	165.52	3.84	0.0025				
	JA	LOC_Os03g08330	8.20	83.43	3.35	0.0091	13.30	175.76	3.72	0.0025	6.11	44.52	2.87	0.0067

	JA	LOC_Os03g28940					41.94	267.28	2.67	0.0025	29.57	93.94	1.67	0.0067
	JA	LOC_Os10g25230	0.52	53.10	6.67	0.0091	1.99	70.03	5.14	0.0025	0.51	13.07	4.68	0.0485
	JA	LOC_Os10g25290					13.20	145.74	3.46	0.0025				
Phytohormone biosynthesis	Auxin	LOC_Os04g45720					1.55	0.20	-2.93	0.0159				
	GA	LOC_Os03g63970									32.18	2.02	-3.99	0.0067
	GA	LOC_Os01g66100									5.40	0.42	-3.67	0.0067
	GA	LOC_Os02g36140					0.22	24.12	6.78	0.0025				
	GA	LOC_Os04g09900	0.07	2.96	5.33	0.0165	0.84	26.60	4.98	0.0025				
	ABA	LOC_Os12g43130					0.45	2.28	2.35	0.0261	0.56	12.40	4.47	0.0067
	JA	LOC_Os03g32314					56.28	226.88	2.01	0.0025				
	JA	LOC_Os06g11210					36.76	470.54	3.68	0.0025	16.03	80.69	2.33	0.0067

Table S6. Expression profiles of putative phytochrome genes.

Names	Genes	20 minutes				60 minutes				360 minutes			
		Dark (FPKM)	Light (FPKM)	Light/Dark	p_value	Dark (FPKM)	Light (FPKM)	Light/Dark	p_value	Dark (FPKM)	Light (FPKM)	Light/Dark	p_value
OsphyA	LOC_Os03g51030									54.623	22.804	0.42	0.003
OsphyB	LOC_Os03g19590					11.174	8.691	0.78	0.004				
OsphyC	LOC_Os03g54084												
OsCRY1a	LOC_Os02g36380	10.346	6.299	0.61	0.037	10.673	6.684	0.63	0.003				
OsCRY1b	LOC_Os04g37920	1.647	0.617	0.37	0.047								
OsPIL11	LOC_Os12g41650												
OsPIL12	LOC_Os03g43810												
OsPIL13	LOC_Os03g56950												
OsPIL14	LOC_Os07g05010									2.396	8.292	3.46	0.023
OsPIL16	LOC_Os05g04740												

Table S7. Primers used for the real-time PCR analysis that was compared with the RNA-Seq analysis.

Phytohormones	Genes	Forward primer（5'- 3'）	Reverse primer（5'- 3'）
Auxin	LOC_Os01g12160	CTCGTCAAGTCCACGATGCA	CCACCGGAAACTCCCTGTT
GA	LOC_Os01g66100	GCCGACTACTTCTCCAGCACCC	GCTGTCCGCGAAGAACTCCCT
	LOC_Os08g33660	ACACCAAGCAAACTCCATGCACAG	AGTTCATCGCTTCTTTGCTGCCAC
JA	LOC_Os03g08310	AATGGCCTTGAGTCGGTAGATC	CCCCATAGGAATCGAAAAAAAA
	LOC_Os03g28940	TTGATGACTTCCCAGCTGAGAA	GCGCTGTGGAGGAACTCTTG
	LOC_Os03g32314	TGCCTCAACAACTTCACCAACTA	CACATGCCGCAATTAACACTAAA
	LOC_Os05g50890	CCAAGGAAAAGCACTGCAGTT	GCAAGAATGCCACCTTTCGT
	LOC_Os10g25230	GCACGATTATTCCTATCTGTCACAA	TCCATCATCGTTCTTTCAGTATTTTT

Fig. S1. Comparison of qRT-PCR (blue bar) and RNA-seq (Red bar) data for selected gene.

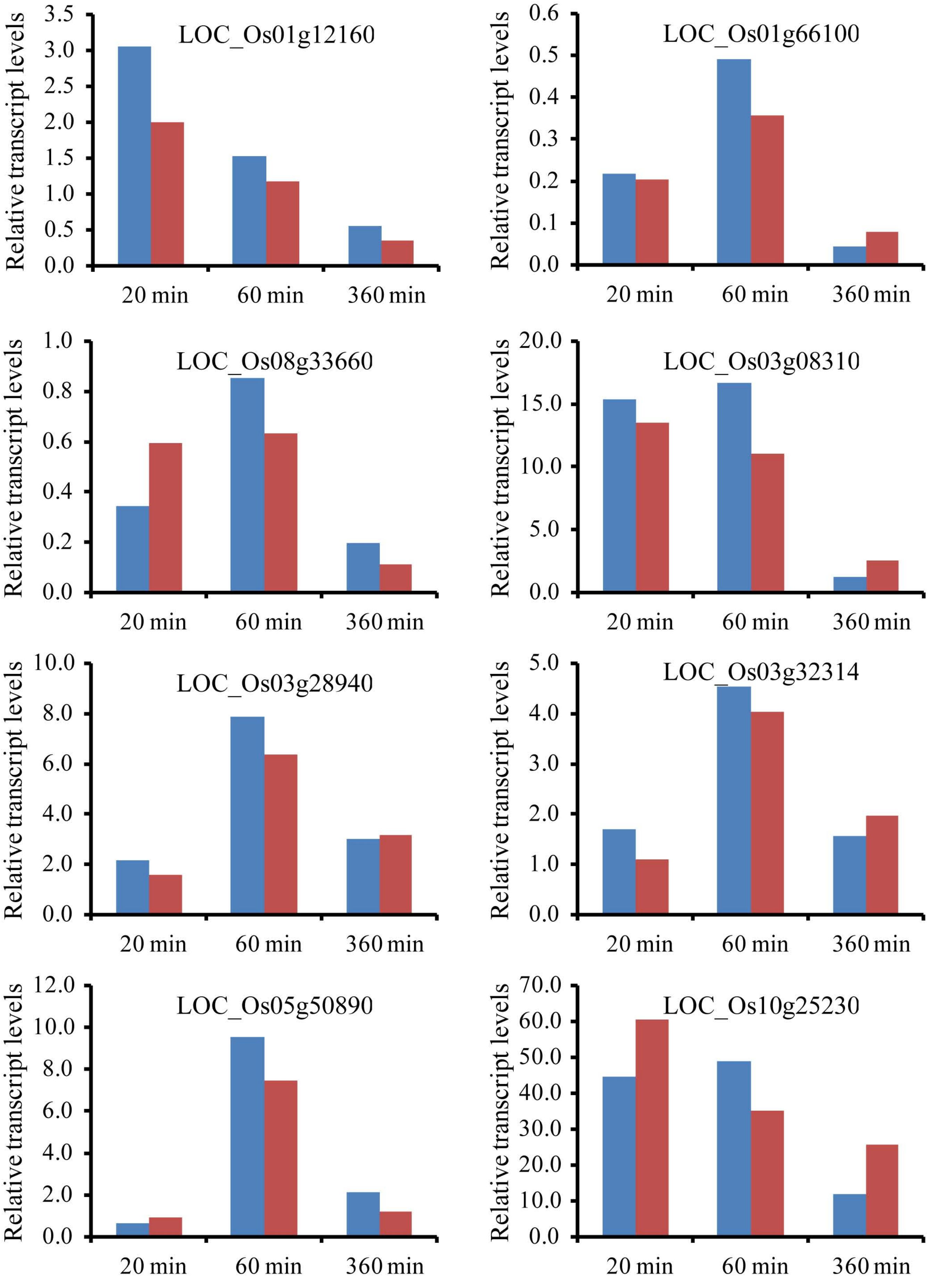

Fig. S2. The effects of exogenous phytohormones (IAA, tZ, GA_3, ABA and JA) on mesocotyl elongation of the etiolated rice seedlings grown for 2 d after germination in dark. (A) Phenotype of etiolated seedlings treated respectively with exogenous phytohormones. Arrowheads indicate positions of the coleoptilar nodes between mesocotyl and coleoptile. (B) Mesocotyl length of etiolated seedlings treated respectively with exogenous phytohormones. The values are means ±SD of at least 10 seedlings per treatment.The asterisks indicate significant difference compared with CK (**P < 0.01, Student's *t* test).

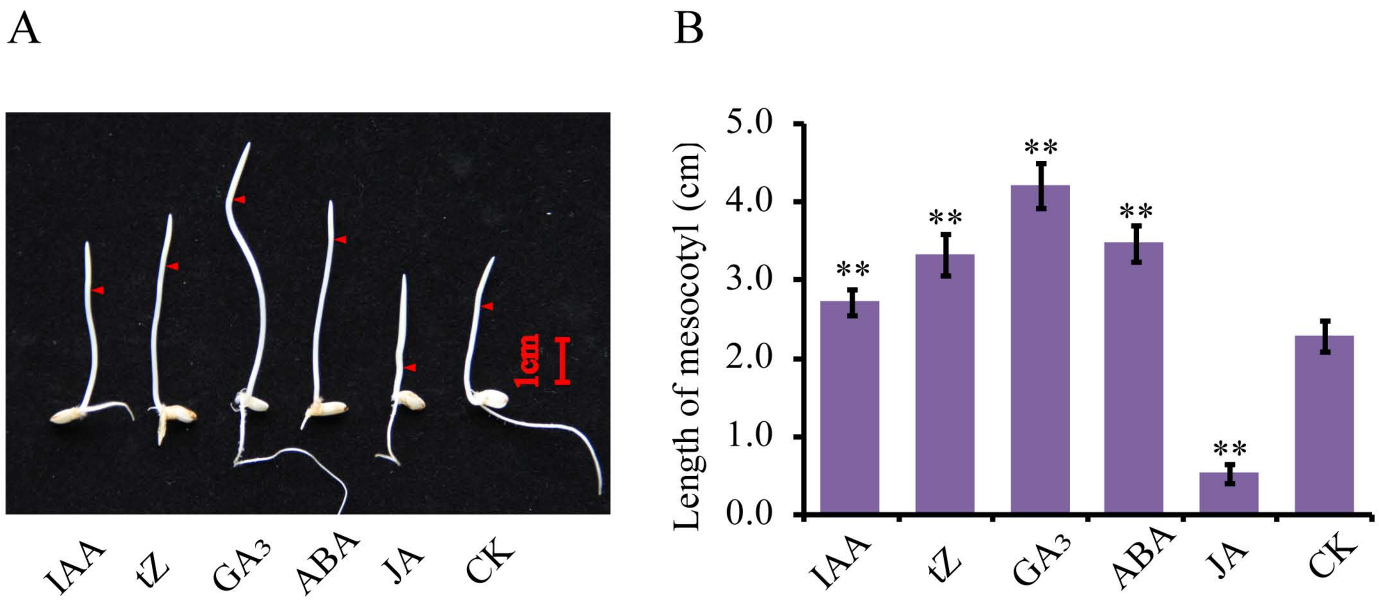

Supplement: Supplementary file 1 — Supplementary tables and figures [file 41598_2017_12326_MOESM1_ESM.pdf]
